# Comparative genomics of host adaptive traits in *Xanthomonas translucens* pv. *graminis*

**DOI:** 10.1186/s12864-016-3422-7

**Published:** 2017-01-05

**Authors:** Lena Hersemann, Daniel Wibberg, Jochen Blom, Alexander Goesmann, Franco Widmer, Frank-Jörg Vorhölter, Roland Kölliker

**Affiliations:** 1 Molecular Ecology, Agroscope, 8046 Zurich, Switzerland; 2 Center for Biotechnology, Bielefeld University, 33615 Bielefeld, Germany; 3 Bioinformatics and Systems Biology, Justus Liebig University Giessen, 35392 Giessen, Germany; 4 MVZ Dr. Eberhard & Partner Dortmund, 44137 Dortmund, Germany

**Keywords:** Inter-pathovar comparison, Type VI secretion system, LPS O-antigen, HrpE, PilA

## Abstract

**Background:**

*Xanthomonas translucens* pathovars differ in their individual host ranges among Poaceae. As the causal agent of bacterial wilt in Italian ryegrass (*Lolium multiflorum* Lam.), *X. translucens* pv. *graminis* (*Xtg*) is one of the most important bacterial pathogens in temperate grassland regions. The genomes of six *Xtg* strains from Switzerland, Norway, and New Zealand were sequenced in order to gain insight into conserved genomic traits from organisms covering a wide geographical range. Subsequent comparative analysis with previously published genome data of seven non-*graminis X. translucens* strains including the pathovars *arrhenatheri*, *poae*, *phlei*, *cerealis*, *undulosa*, and *translucens* was conducted to identify candidate genes linked to the host adaptation of *Xtg* to Italian ryegrass.

**Results:**

Phylogenetic analysis revealed a tight clustering of *Xtg* strains, which were found to share a large core genome. Conserved genomic traits included a non-canonical type III secretion system (T3SS) and a type IV pilus (T4P), which both revealed distinct primary structures of the pilins when compared to the non-*graminis X. translucens* strains. *Xtg*-specific traits that had no homologues in the other *X. translucens* strains were further found to comprise several hypothetical proteins, a TonB-dependent receptor, transporters, and effector proteins as well as toxin-antitoxin systems and DNA methyltransferases. While a nearly complete flagellar gene cluster was identified in one of the sequenced *Xtg* strains, phenotypic analysis pointed to swimming-deficiency as a common trait of the pathovar *graminis*.

**Conclusion:**

Our study suggests that host adaptation of *X. translucens* pv. *graminis* may be conferred by a combination of pathovar-specific effector proteins, regulatory mechanisms, and adapted nutrient acquisition. Sequence deviations of pathogen-associated molecular patterns (PAMPs), as observed for the pilins of the T4P and T3SS, are moreover likely to impede perception by the plant defense machinery and thus facilitate successful host colonization of Italian ryegrass.

**Electronic supplementary material:**

The online version of this article (doi:10.1186/s12864-016-3422-7) contains supplementary material, which is available to authorized users.

## Background

Grasslands and pastures cover the largest agricultural area worldwide and serve as the prevalent source of roughage for ruminants in meat and milk production [1]. Bacterial wilt of forage grasses, caused by the bacterium *Xanthomonas translucens* pv. *graminis* (*Xtg*), is a serious issue in temperate grassland regions [2]. In the field, contaminated mowing tools are considered to be the primary source of inoculation and infected plants are easy to recognize by the characteristic withering of leaves and tillers as well as yellow stripes, occasionally occurring along the leaf blades [3]. Highly susceptible plants have been found to die within three weeks after inoculation, whereas less susceptible plants suffer from reduced regrowth and deformed inflorescences [2, 3]. *Xtg* is characterized by a wide host range, including species of the grass genera *Lolium*, *Phleum*, *Festuca*, *Dactylis*, and *Trisetum* [4]. Most pronounced, economic impact has been described for *Xtg* infections of Italian ryegrass (*Lolium multiflorum*), which represents an important source of fodder in dairy production [5]. With view on the high yield losses observed across infected plants, *Xtg* resistance is of major concern for breeding of new Italian ryegrass cultivars. The currently applied approach is based on recurrent phenotypic selection; however, reoccurrence of susceptible individuals after several selection cycles indicates the need for a detailed understanding of underlying pathogenicity mechanisms, which can be exploited in targeted breeding for bacterial wilt resistance [6, 7].

Pathogenic bacteria rely on virulence factors to (i) enter the host plant, (ii) to colonize it, and (iii) to overcome plant defense mechanisms. The latter are triggered by the perception of pathogen-associated molecular patterns (PAMPs) like flagellin and lipopolysaccharides (LPS), which can lead to the accumulation of antimicrobial substances and the release of reactive oxygen species [8, 9]. To counteract these defense mechanisms, many bacteria rely on a type III secretion system (T3SS) in order to secrete effector proteins, which can interfere with plant cellular processes in favor of the pathogen [10]. It has been shown for *X. campestris* pv. *vesicatoria* that a functional T3SS is important for successful plant colonization and symptoms development [11]. Genome sequencing of the *X. translucens* pv. *graminis* strain Xtg29 revealed the presence of a non-canonical type III secretion system with deviations in gene order and sequence homology in comparison to the classical T3SS, which is well conserved among other *Xanthomonas* species [12]. Site-directed knockout mutagenesis of the main regulator HrpG and the structural components HrpE and HrcR in Xtg29 impaired bacterial virulence on Italian ryegrass, but the tested mutants were not affected in *in planta* colonization [12]. Therefore, it is likely that *Xtg* possesses further virulence factors, which crucially influence its pathogenicity on Italian ryegrass. Genome sequencing has long become the prevalent tool for gaining insight into putative virulence factors of plant pathogenic bacteria. The main objective of this study was the identification of virulence factors which enable *Xtg* to cause bacterial wilt in Italian ryegrass. We sequenced six *Xtg* strains collected in Switzerland, Norway, and New Zealand in order to identify the common core genome and shared virulence-related traits, which are conserved among *Xtg* strains from a wide geographical range. As many virulence-contributing factors represent host-independent, common characteristics of plant pathogenic bacteria [13], we were further aiming to identify host adaptive traits by comparing the *Xtg* core genome with closely related strains, which are non-pathogenic on Italian ryegrass. For this, we exploited the close genetic relation of *Xtg* with other recently sequenced *X. translucens* strains with pathogenicity on either forage grasses (i.e. *X. t.* pv. *arrhenatheri* LMG 727, *X. t.* pv. *poae* LMG 728, and *X. t.* pv. *phlei* LMG 730) or cereals (i.e. *X. t.* pv. *cerealis* CFBP 2541, *X. t.* pv. *undulosa* Xtu4699, *X. t.* pv. *translucens* DSM 18974, and *X. t.* DAR61454) [14–18]. In contrast to *Xtg*, the other three forage grass affecting pathovars are characterized by high host specialization to the plant species from which they have originally been isolated, i.e. *Arrhenatherum elatius*, *Poa trivialis*, and *Phleum pratense* [4]. The cereals affecting *X. translucens* strains, in turn, have primarily been described for their pathogenicity on barley, rye, and wheat [18, 19]. Due to their close genetic relation and the distinct host specificity, the *X. translucens* pathovars represent an excellent system for studying host adaptation at the genome level.

## Methods

### Strains, growth conditions, and DNA extraction

Six *Xanthomonas translucens* pv. *graminis* (*Xtg*) strains were sequenced in this study (Table 1). The four Swiss strains were part of recent studies on genetic diversity of pv. *graminis* isolates from Switzerland [20]. Based on amplified fragment length polymorphism (AFLP) analyses, 28 out of 30 investigated *Xtg* isolates have been found to cluster into two major groups, which were significantly influenced by the geographic location of the corresponding sampling site. The Swiss strains were chosen as representatives of cluster I (Xtg9 and Xtg29), cluster II (Xtg2), and of strains not grouping in clusters (Xtg10). Two additional *Xtg* strains isolated in Norway (NCPPB 3709) and New Zealand (ICMP 6431) were obtained as freeze dried cultures from the National Collection of Plant Pathogenic Bacteria (Fera Science Ltd., Sand Hutton, United Kingdom) and the International Collection of Microorganisms from Plants (Landcare Research, Auckland, New Zealand), respectively. Unless otherwise stated, bacteria were cultivated at 28 °C on either GYC agar medium [20] or in 3 ml CircleGrow® broth (MP Biomedicals, Santa Ana, USA) in 15 ml Falcon™ tubes (BD, New Jersey, USA) and by shaking at 200 rpm. For DNA extraction, the CTAB method [21] was used by applying modifications as described recently [16].

**Table 1:** *X. t.* pv. *graminis* strains used for whole genome sequencing

Strain	Host plant	Isolation site	Origin or reference
Xtg29	*Lolium multiflorum*	Switzerland (Changins)	[20]
Xtg2	*Lolium multiflorum*	Switzerland (Beinwil)	[20]
Xtg9	*Lolium multiflorum*	Switzerland (Ellighausen)	[20]
Xtg10	*Lolium multiflorum*	Switzerland (Ellighausen)	[20]
NCPPB 3709	*Lolium perenne*	Norway	NCPPB^a^
ICMP 6431	*Lolium perenne*	New Zealand	ICMP^b^

^a^ *NCPPB* National Collection of Plant Pathogenic Bacteria

^b^ *ICMP* International Collection of Microorganisms from Plants

### Genome sequencing

Whole genome sequencing was conducted for all *Xtg* strains listed in Table 1. In view of the sequencing costs, homopolymer resolution, and observed GC bias in library preparations [22, 23], we decided to apply the Illumina MiSeq System by sequencing (2 × 250 bp) paired-end libraries constructed with the TrueSeq DNA LT Sample Prep Kit (Illumina Inc., San Diego, United States). The Swiss strain Xtg29, which has previously been sequenced by applying the Roche 454 Genome Sequencer FLX System [12], was re-sequenced in order to prevent bias in comparative genome analysis due to deviating sequencing strategies. Genome assembly of Xtg29 was moreover facilitated by (2 × 250 bp) sequencing of an additional 8 kb mate-pair library on an Illumina MiSeq System (Illumina Inc., San Diego, United States). For the following comparative genome analysis, we used Xtg29 as our reference strain for *Xtg*.

### Assembly and annotation

After sequencing, the raw data was processed with an in-house pipeline including CASAVA version 1.8 as described recently [24]. The *de novo* genome assembly of all strains was conducted with the GS de Novo Assembler software version 2.8 with default settings (Roche, Basel, Switzerland). Genomes were annotated using an in-house pipeline based on the workflow engine Conveyor [25]. This pipeline uses Prodigal [26], RNAMMER [27] and ARAGORN [28] for the prediction of coding sequences, rRNAs, and tRNAs. Functional annotation is realized by a comparison of predicted coding sequences against a database of conserved orthologous groups of the genus *Xanthomonas* identified by the EDGAR software [29]. Genes that could not be annotated by this approach were compared to a collection of sequence databases comprising Swissprot, Refseq, and Pfam-A [30–32].

### Genome comparisons

Publically available genome data of seven non-*graminis X. translucens* strains were used for comparative genome analysis (Table 2). In order to facilitate data comparability, the assemblies of all non-*graminis* strains were annotated *de novo* by using the same strategy as described above. Assignments of the four forage grass affecting *X. translucens* pathovars (i.e. pv. *arrhenatheri*, pv. *graminis*, pv. *poae*, and pv. *phlei*) to the *graminis* group followed the denomination by Vauterin et al. [33].

**Table 2:** *Xanthomonas* genomes used for comparative genome analysis

Strain^a^	Accession number	Reference
*X. translucens* pv. *graminis* Xtg29	PRJEB10857	this study
*X. translucens* pv. *graminis* Xtg2	PRJEB10858	this study
*X. translucens* pv. *graminis* Xtg9	PRJEB10859	this study
*X. translucens* pv. *graminis* Xtg10	PRJEB10860	this study
*X. translucens* pv. *graminis* NCPPB 3709	PRJEB10862	this study
*X. translucens* pv. *graminis* ICMP 6431	PRJEB10861	this study
*X. translucens* pv. *graminis* CFBP 2053^PT^	PRJNA290469	[47]
*X. translucens* pv. *arrhenatheri* LMG 727^PT^	PRJEB9902	[16]
*X. translucens* pv. *poae* LMG 728^PT^	PRJEB9904	[16]
*X. translucens* pv. *phlei* LMG 730^PT^	PRJEB9905	[16]
*X. translucens* pv. *cerealis* CFBP 2541^PT^	PRJNA268946	[14]
*X. translucens* pv. *undulosa* Xtu4699	PRJNA248137	[15]
*X. translucens* pv. *translucens* DSM 18974^T^	PRJEB647	[17]
*X. translucens* DAR61454	PRJNA169523	[18]
*X. oryzae pv. oryzae* PXO99A	PRJNA28127	[82]

^a^ Pathotype strains (^PT^) and the *X. translucens* type strain (^T^) are indicated

Comparative genome analysis was performed using EDGAR 2.0 [29]. The platform employs a bit score related cutoff value, which is generated based on BLAST score ratio values (SRVs) to identify orthologous genes. Thus, coding sequences (CDS) from different genomes are considered orthologous, if the predicted SRV exceeds the calculated cutoff. This approach allows the identification of real orthologues as representatives of the calculated core genomes. In contrast, only CDS without any hit against any of the other genomes are considered as true singletons [34]. EDGAR 2.0 was used for the calculation of core and pan genomes across the *X. translucens* strains and true singleton prediction as well as for the construction of a core genome based phylogenetic tree. Therefore, coding sequences conserved among all analyzed genomes were aligned using MUSCLE [35], concatenated, and used to create a phylogeny using Kimura distances and the neighbor-joining method as implemented in the PHYLIP package [36]. Additionally, average nucleotide identities (ANI) of the orthologous genes of the core genome [37] were calculated using EDGAR 2.0.

### Singleton and gene cluster analysis

Identification and characterization of the virulence-contributing gene clusters and the predicted singletons was conducted by a combined approach of BLASTP analysis [38], conserved domain search [39], and signal peptide prediction by SignalP 4.0 [40]. Moreover, plant-inducible promoter (PIP) boxes [41] were identified by regular expression search as described recently [12]. Class III signal peptides in type IV pilin sequences were identified based on the core motif [GAS]-[ACFGILMNPQSTVWY]4-[DE] as reported by Imam et al. [42]. Taking the obtained results into account, annotation of the gene region and/or gene function was manually revised when considered appropriate. Analysis of the assembly gap observed for two neighboring, predicted singletons of the *Xtg* core genome was done using Sanger sequencing. Therefore, we amplified the gap-spanning region in 20 μl reaction volumes adjusted with ddH_2_O and containing 20% (*v*/*v*) 5 × GC buffer, 3% (*v*/*v*) DMSO, 0.05 mM of each dNTP, 0.2 μM forward primer (AGGCGTGTGGAAACGCACTG), 0.2 μM reverse primer (CCAGTGGCGTGATCTTCACC), 50 ng genomic DNA of Xtg29, and 0.4 U Phusion Hot Start II DNA polymerase (Thermo Fisher Scientific, Waltham, USA). Thermocycler conditions were set to initial denaturation for 1 min at 98 °C, 30 cycles of denaturation for 10 s at 98 °C, annealing for 10 s at 64 °C, and elongation for 15 s at 72 °C, followed by one final elongation for 7 min at 72 °C. The obtained PCR product was purified with the NucleoSpin® Gel and PCR Clean-up kit (Macherey-Nagel, Düren, Germany). Sequencing was performed with the the BigDye® Terminator v3.1 Cycle Sequencing Kit and the ABI Prism 3130xl Genetic Analyzer following the manufacturer’s recommendations (Applied Biosystems, Foster City, USA).

### Pathogenicity assay

All six *Xtg* strains listed in Table 1 were verified for pathogenicity on the Italian ryegrass (*Lolium multiflorum*) genotype LmK-01 [43] using four weeks old, clonally propagated plants with five to ten tillers each. The bacteria were grown over night in liquid medium containing 2% (*w*/*v*) glucose and 0.5% (*w*/*v*) yeast extract at 28 °C and 200 rpm. Cells were harvested by centrifugation for 5 min at 6500 g, resuspended in 0.8% (*w*/*v*) sodium chloride and diluted to a final density of OD_580nm_ = 0.3, corresponding to 9 × 10^8^ colony forming units per ml. Inoculation was conducted by cutting the plants with scissors dipped into the bacterial suspensions. As a negative control, sterile aqueous 0.8% (*w*/*v*) sodium chloride solution was used. Per treatment, four pots with one plant each were inoculated. Assessment of symptoms development was performed 28 days post inoculation (dpi) and was based on a scoring system of nine indices representing disease severity in ascending order from no symptoms (1), over quantitative increasing wilting symptoms on leaves and tillers to finally dead plants (9) [43]. Analysis of variance and predefined contrasts implemented in R statistical software [44] were used to test for significant differences between the control treatment and the bacterial strains.

### Motility assay

Motility assays were performed on TY agar medium containing 0.5% (*w*/*v*) tryptone, 0.3% (*w*/*v*) yeast extract, 0.07% (*w*/*v*) calcium chloride, and 0.3% (*w*/*v*) BactoTM Agar (Becton, Dickinson and Company, Sparks, USA). Bacterial colonies, grown on solid GYC agar medium, were transferred by a toothpick to the center of the TY agar surface. Spreading of the individual colonies was photographically documented after ten days incubation time at 28 °C.

## Results

### Genome sequencing of *X. t.* pv. *graminis* strains

Whole genome sequence data were determined for six *Xtg* strains (Xtg29, Xtg2, Xtg9, Xtg10, NCPPB 3709, and ICMP 6431), as a prerequisite for subsequent comparative analysis (Additional file 1: Table S1). The obtained number of contigs (>500 bp) varied from 349 for ICMP 6431 to 369 for Xtg29 with an average N50 contig length of 18,459 bp. Genome data based on sequencing of single paired-end libraries only, revealed 284 to 296 scaffolds, while the combination with mate-pair sequencing data, as applied for Xtg29, reduced the number of scaffolds to three. In contrast, a previous Xtg29 assembly had 788 contigs (> 500 bp) and 12 scaffolds upon sequencing with the 454 FLX System [12]. *De novo* annotation of the 454 assembly by using the same strategy as applied in the present study, revealed 3619 predicted coding sequences (CDS) while 3543 CDS were identified for the Illumina MiSeq-based assembly of the re-sequenced Xtg29 genome (Additional file 2: Table S2). Differing annotation and assembly parameter reflect the technological progress manifest in current Illumina sequencing techniques compared to previous 454-based methods.

### *Xtg* strains differed in their genome size from other *X. translucens* pathovars

All seven *Xtg* strains were characterized by a reduced genome size when compared against the non-*graminis X. translucens* strains (Table 3). The six *Xtg* strains, sequenced in this study, further revealed a distinctly smaller number of predicted CDS (3487 to 3553 CDS) in comparison to 3776 to 3957 CDS predicted for the other *X. translucens* strains including the pv. *graminis* pathotype strain CFBP 2053.

**Table 3:** Genome features of *X. translucens* strains

Strain^a^	*X. t.* pv. *graminis* Xtg29	*X. t.* pv. *graminis* Xtg2	*X. t.* pv. *graminis* Xtg9	*X. t.* pv. *graminis* Xtg10	*X. t.* pv. *graminis* NCPPB 3709	*X. t.* pv. *graminis* ICMP 6431	*X. t.* pv. *graminis* CFBP 2053^PT*^	*X. t.* pv. arrhenatheri LMG 727^PT*^	*X. t.* pv. *poae* LMG 728^PT*^	*X. t.* pv. *phlei* LMG 730^PT*^	*X. t.* pv. *cerealis* CFBP 2541^PT*^	*X. t.* pv. *undulosa* Xtu4699^*^	*X. t.* pv. *translucens* DSM 18974^T*^	*X. t.* DAR61454*
Attribute														
Genome size	4.2 Mb	4.2 Mb	4.1 Mb	4.2 Mb	4.2 Mb	4.1 Mb	4.3 Mb	4.8 Mb	4.6 Mb	4.4 Mb	4.5 Mb	4.6 Mb	4.7 Mb	4.5 Mb
Protein coding genes	3543	3529	3519	3529	3553	3487	3851	3871	3845	3744	3888	3835	3957	3776
Genes with function prediction	2328	2323	2317	2326	2367	2297	2572	2531	2505	2477	2466	2540	2586	2439
Genes with Pfam domains	2795	2795	2788	2789	2825	2749	3069	3062	3009	2980	2965	3055	3091	2907
Genes with transmembrane helices	877	876	866	867	887	865	936	953	933	918	910	894	951	851
Genes with signal peptides	549	551	555	554	551	545	553	584	584	552	521	531	563	521

^a^ Pathotype strains (^PT^) and the *X. translucens* type strain (^T^) are indicated. Data obtained by using the same annotation strategy as applied for the six *Xtg* strains (Xtg29, Xtg2, Xtg9, Xtg10, NCPPB 3709 and ICMP 6431) sequenced in this study are indicted by asterisks (*)

### Inter-pathovar phylogeny of the species *X. translucens* revealed a close genetic relation of *Xtg* strains

**Fig. 1:**
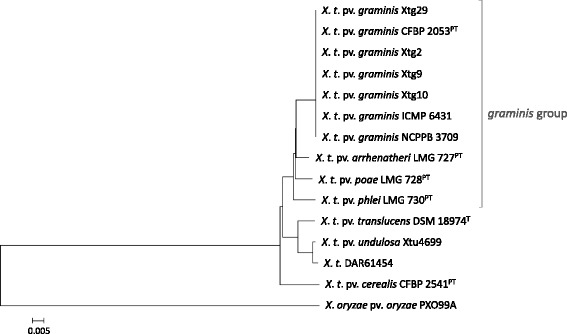
Phylogeny of *X. translucens* pathovars. The calculated phylogenetic tree was based on the amino acid sequences of CDS conserved among 15 genomes from seven pv. *graminis* strains, seven non-*graminis X. translucens* strains, and *X. oryzae* pv. *oryzae* PXO99A as an outgroup. Pathotype strains (^PT^) and the *X. translucens* type strain (^T^) are indicated. In total, 32,220 coding sequences (2148 per genome) with 11,474,880 amino acid residues (764,992 per genome) were used for the construction of the tree using the neighbor-joining method. In 500 iterations all branches showed at least 56.2% bootstrapping support (Additional file 11: Figure S6)

Phylogenetic analysis was carried out based on the amino acid (AA) sequences of orthologous CDS conserved among the different *X. translucens* pathovars, using the rice pathogen *X. oryzae* pv. *oryzae* PXO99A as an outgroup. In the resulting phylogenetic tree, the four forage-grass affecting *X. translucens* pathovars of the *graminis* group were separated from cereals-affecting *X. translucens* strains (Fig. 1). Furthermore, we found all *Xtg* strains including the pathotype strain CFBP 2053 to cluster tightly together. Across those, no distinct intra-pathovar differences were observed.

**Fig. 2:**
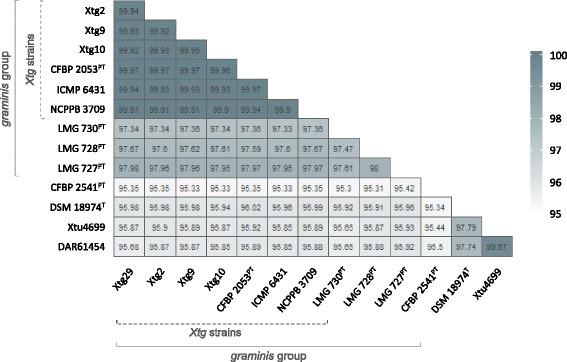
Average nucleotide identities of *X. translucens* pathovars. The data represent the mean percentage identities of orthologues shared by seven pv. *graminis* strains (i.e. Xtg29, Xtg2, Xtg9, Xtg10, ICMP 6431, NCPPB 3709, and CFBP 2053) as well as seven non-*graminis* strains representing different *X. translucens* pathovars (i.e. *X. t.* pv. *arrhenatheri* LMG 727, *X. t.* pv. *poae* LMG 728, *X. t.* pv. *phlei* LMG 730, *X. t.* pv. *undulosa* Xtu4699, *X. t.* pv. *cerealis* CFBP 2541, *X. t.* pv. *translucens* DSM 18974, and *X. t.* DAR61454). Pathotype strains (^PT^) and the *X. translucens* type strain (^T^) are indicated

Similar results were observed for the calculated average nucleotide identities (ANI). Thus, the forage-grass affecting *X. translucens* pathovars, i.e. pv. *arrhenatheri*, pv. *poae*, pv. *phlei*, and pv. *graminis* shared ANI values of 97.33 to 98% when compared among each other, but revealed only 95.3 to 95.99% ANI with the cereals-affecting *X. translucens* strains (Fig. 2). While also the non-*graminis* strains *X. t.* pv. *translucens* DSM 18974, *X. t.* pv. *undulosa* Xtu4699, and *X. t.* DAR61454 were found to possess ANI values of 97.74 to 99.67%, highest average nucleotide identities of 99.9 to 99.97% were observed for the seven *Xtg* strains.

### All *Xtg* strains caused bacterial wilt on Italian ryegrass (*Lolium multiflorum*)

**Fig. 3:**
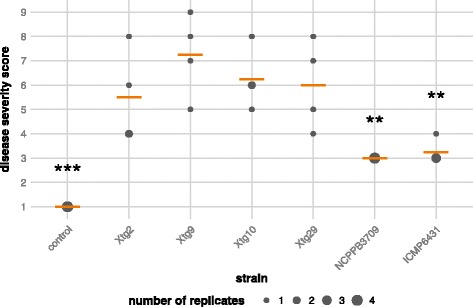
Pathogenicity of forage grass affecting *X. translucens* pv. *graminis* strains on Italian ryegrass (*Lolium multiflorum*). Disease severity scores for bacterial wilt ranging from no symptoms (*1*) to dead plants (*9*) were evaluated 28 days post infection for four replicates per tested strain. Size of *dots* indicates the number of replicates allocated to a particular score, each represented by one clonal replicate of the *L. multiflorum* genotype LmK-01. *Orange bars* indicate the mean over the four replicates. The six tested strains of *Xtg* originated from different geographical locations, i.e. Switzerland (Xtg2, Xtg9, Xtg10, and Xtg29), Norway (NCPPB 3709) and New Zealand (ICMP 6431). *Asterisks* indicate significant differences in comparison to Xtg29 (*** *p* < 0.001; ** *p* < 0.01)

Prior to detailed genome-based comparison, we analyzed the *Xtg* strains sequenced in this study for their pathogenicity on *Lolium multiflorum* (*Lm*). Clear wilting symptoms were found on all *Lm* plants inoculated with any of the six *Xtg* strains four weeks after clipping inoculation (Fig. 3); however, disease severity varied between the individual strains. Most pronounced symptoms with average wilting scores of 5.5, 7.25, 6.25, and 6 were observed for the four Swiss strains, i.e. Xtg2, Xtg9, Xtg10, and Xtg29. In contrast, only moderate symptoms were found for the two strains collected in Norway (NCPPB 3709) and New Zealand (ICMP 6431), reflected by average scores of 3 and 3.25, respectively. It is noticeable, that replicates of the less aggressive strains showed clearly lower variance of at most one score difference than observed for the Swiss strains, which varied over a range of up to four scores. Nevertheless, variance analysis indicated statistically significant differences in virulence (*p* < 0.01) between the Swiss strain Xtg29 and NCPPB 3709 and ICMP 6431, respectively.

With regard to the detected differences in virulence, we performed whole genome comparison of the *Xtg* strains from different geographical locations. However, we did not find any orthologues shared by the Swiss strains (i.e. Xtg29, Xtg2, Xtg9, and Xtg10) but absent in the strains isolated in Norway (NCPPB 3709) and New Zealand (ICMP 6431), respectively (Additional file 3: Figure S1). Likewise, no CDS were found to be exclusively conserved in NCPPB 3709 and ICMP 6431.

### Intra-pathovar comparison of *Xtg* strains revealed a flagellar gene cluster and a type VI secretion system as strain-specific traits

**Fig. 4:**
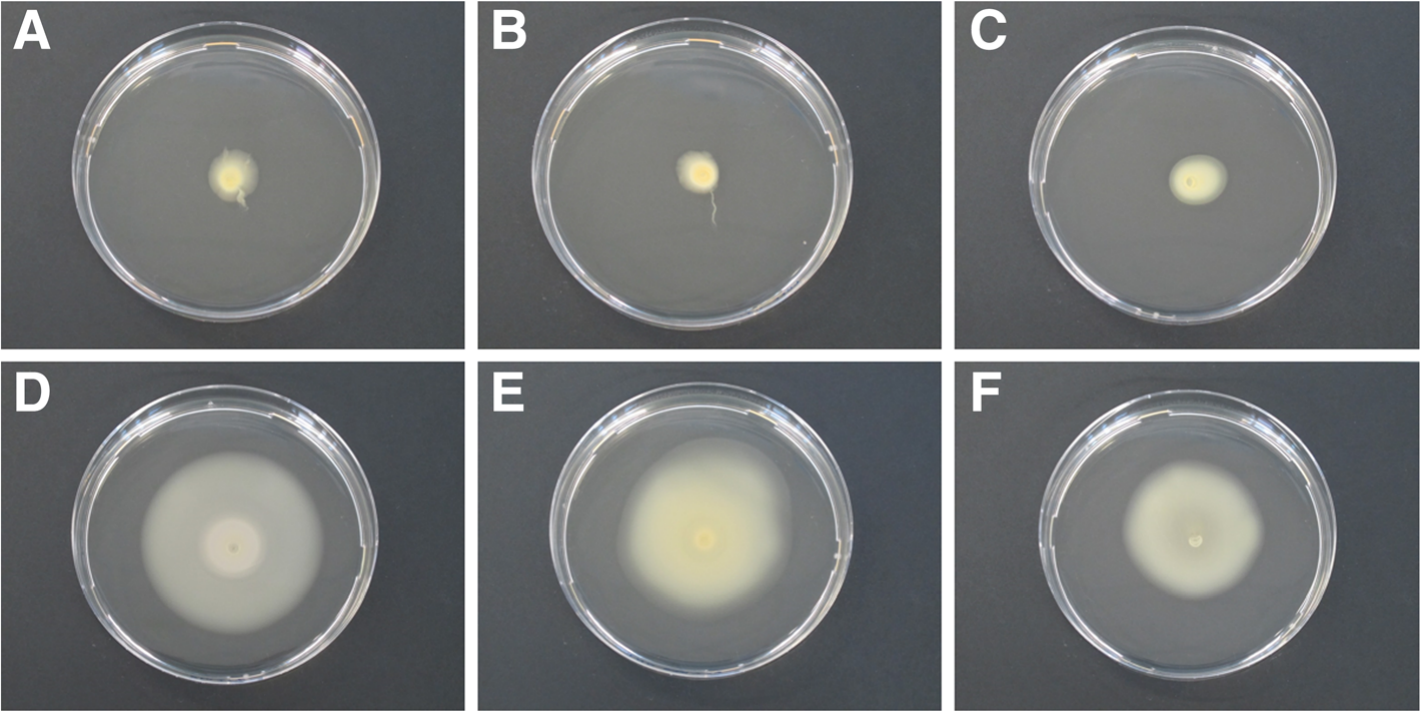
Motility assay of *X. translucens* pathovars on soft agar. Absence and presence of flagellar-mediated swimming motility of three *X. t.* pv. *graminis* strains, i.e. Xtg29 (**a**), NCPPB 3709 (**b**), and ICMP 6431 (**c**) as well as *X. t.* pv. *arrhenatheri* LMG 727 (**d**), *X. t.* pv. *poae* LMG 728 (**e**), and *X. t.* pv. *phlei* LMG 730 (**f**) after incubation for 10 days at 28 °C

**Fig. 5:**
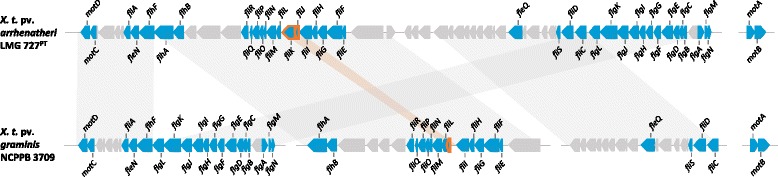
Comparison of the flagellar gene cluster of *X. t.* pv. *arrhenatheri* LMG 727 and *X. t.* pv. *graminis* NCPPB 3709. Without *motA* and *motB*, the illustrated flagellar gene cluster of LMG 727 spanned a region of 68.77 kb comprising 65 coding sequences. *Grey patches* emphasized the observed rearrangement of the corresponding gene cluster in NCPPB 3709. Genes encoding for structural components of the flagellum (*fli*, *fle, flg*, *flh*) and components of the motor complex, i.e. *motA*, *motB*, *motC*, and *motD* are illustrated by *blue* coloration. Among those, regions with significant differences are highlighted by *orange borders*

For intra-pathovar comparison of the *Xtg* strains, 1 (Xtg10) to 67 (NCPPB 3709) strain-specific CDS were predicted (Additional file 4: Figure S2). With the exception of NCPPB 3709, most of these predicted singletons represented hypothetical proteins with the highest number of 27 CDS observed for the pathotype strain CFBP 2053. This strain was further characterized by nine transposases identified along with 28 identical paralogous genes, while no transposases were present in the singletons predicted for the other *Xtg* strains. The Norwegian strain NCPPB 3709 was found to encode 38 homologues of a flagellar gene cluster and six chemotaxis proteins, which revealed no homologous genes in the other six *Xtg* strains. However, a motility assay on 0.3% agar medium revealed swimming deficiency not just for Xtg29 and ICMP 6431, but also for NCPPB 3709 in comparison to the swimming ability observed for pv. *arrhenatheri*, pv. *poae*, and pv. *phlei* pathotype strains (Fig. 4). Comparison of the flagellar gene cluster of NCPPB 3709 and the pv. *arrhenatheri* pathotype strain LMG 727 revealed the absence of *fliJ* and a truncated *fliK* for the *Xtg* strain (Fig. 5). For Xtg2, two singletons were found to be homologous to genes of a type VI secretion system (T6SS) gene cluster. Additional, 11 T6SS genes, which were identified in the Xtg2 genome, had also homologues in Xtg9, Xtg10 and NCPPB 3709 (Additional file 5: Table S3). For the genomes of Xtg29 and ICMP 6431, no T6SS homologues were observed.

### *Xtg* strains shared a large core genome

All *Xtg* strains were found to share a large common core genome of 3333 CDS. Thus, 86.17% of the predicted pan genome (3868 CDS) was conserved among the seven *Xtg* strains including the pathotype strain CFBP 2053. Various virulence-contributing gene clusters were identified in the *Xtg* core genome, i.e. gene clusters of lipopolysaccharide biosynthesis, xanthan biosynthesis, type I secretion system (T1SS), *xps* type II secretion system (T2SS), the non-canonical type III secretion system (T3SS) as well as the regulatory *rpf* gene cluster of pathogenicity factor synthesis (Table 4). Moreover, we identified 22 homologues of the type IV pilus (T4P) (Additional file 6: Table S4). The major pilin PilA was not predicted by the intrinsic gene prediction tool Prodigal, but analysis of the 1.5 kb DNA region between neighboring *pilC* gene and a putative transposase gene using the ExPASy translate tool predicted a 411 bp gene with a PilA COG domain (E-value: 2.45 × 10^−20^). This gene was further found to possess the characteristic class III signal peptide, which was likewise identified for the minor T4P pilins PilX, PilW, PilV, and FimT. Also PilE revealed a corresponding signal peptide after manual shifting of the translational start site.

**Table 4:** Virulence-related gene clusters identified in *X. t.* pv. *graminis* strains

Gene cluster	Genomes^a^	Reference
type I secretion system	core genome	[83]
type II secretion system	core genome	[84]
type III secretion system, non-canonical	core genome	[12]
type VI secretion system	Xtg2, Xtg9*, Xtg10*, NCPPB 3709*	[51]
type IV pilus	core genome	[59]
flagellum	NCPPB 3709	[85]
regulation of pathogenicity factors	core genome	[86]
xanthan biosynthesis^IPD^	core genome	[87]
lipopolysaccharide biosynthesis^IPD^	core genome	[54]

^a^ The *Xtg* core genome comprises orthologous CDS shared by five Swiss strains, i.e. Xtg2, Xtg9, Xtg10, Xtg29, and CFBP 2053 as well as NCPPB 3709 and ICMP 6431 isolated in Norway and New Zealand, respectively. Asterisks (*) indicate strains whose genomes revealed a reduced number of homologues in comparison to the type VI secretion system gene cluster identified for Xtg2 (Additional file 5: Table S3)

^IPD^ Slight intra-pathovar differences (IPD) of gene clusters conserved among the seven *Xtg* strains were observed for the one encoding for xanthan biosynthesis (gene fusion of *gumK* and *gumL* in the genome of NCBBP 3709) and the gene cluster of LPS biosynthesis (gene separation events due to nonsense mutations in two individual genes among different *Xtg* strains)

While all identified virulence-contributing gene clusters were found to be largely conserved across the seven *Xtg* strains, slight intra-pathovar variations were observed for the gene clusters of xanthan and LPS biosynthesis. Thus, we identified a gene fusion of *gumK* and *gumL* in the *gum* gene cluster of xanthan biosynthesis in the NCPPB 3709 genome. Additionally, the *Xtg* genomes showed slight genomic variance within the putatively O-antigen encoding region of the LPS gene cluster between *etfA* (XTGART29_0580) and *metC* (XTGART29_0559). The observed differences derived from gene separation events due to nonsense mutations in two individual genes.

### Variations in surface exposed structures distinguished *Xtg* strains from other *X. translucens* pathovars

**Fig. 6:**
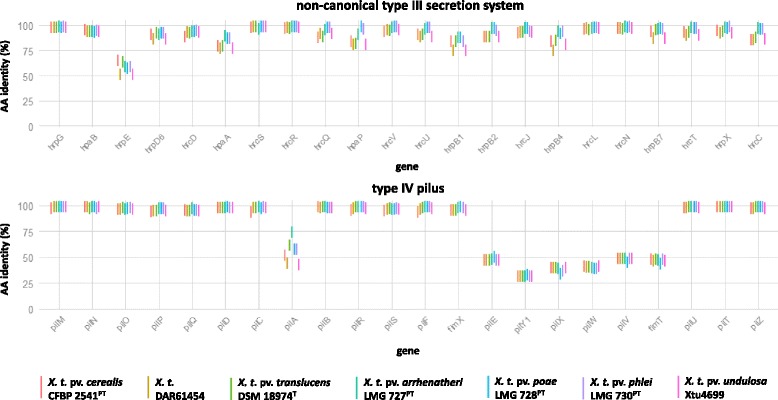
Amino acid sequence identities of T3SS and T4P homologues of non-*graminis X. translucens* strains in comparison to *X. t.* pv. *graminis* Xtg29. Percentage identities of the different *X. translucens* strains (i.e. *X. t.* pv. *arrhenatheri* LMG 727, *X. t.* pv. *poae* LMG 728, *X. t.* pv. *phlei* LMG 730, *X. t.* pv. *cerealis* CFBP 2541, *X. t.* pv. *undulosa* Xtu4699, *X. t.* pv. *translucens* DSM 18974, and *X. t.* DAR61454) were calculated by BLASTP analysis against the identified CDS of the non-canonical type III secretion system and the type IV pilus twitching gene cluster of *X. t.* pv. *graminis* Xtg29. Pathotype strains (^PT^) and the *X. translucens* type strain (^T^) are indicated

**Fig. 7:**
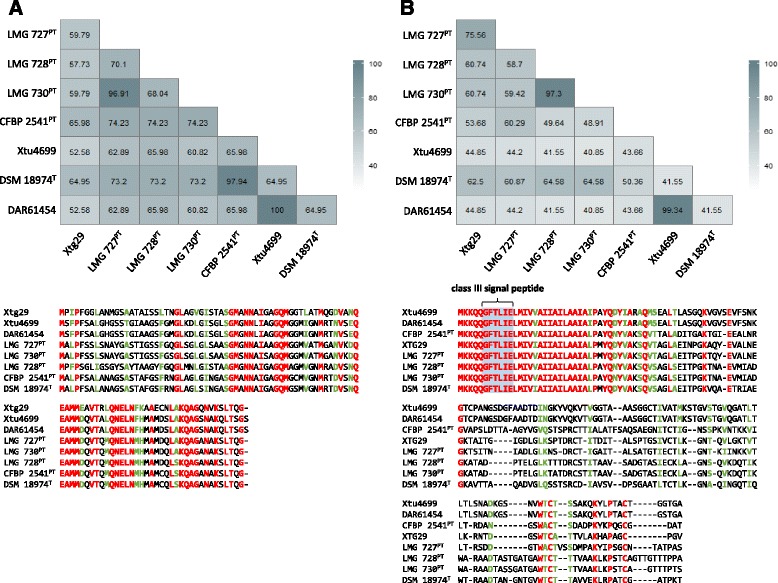
Amino acid sequence similarities of the putative HrpE and PilA homologues of different *X. translucens* pathovars. The data are based on multiple sequence alignments and reflect the percentage identities of the amino acid sequences as well as direct sequence comparison of the HrpE (**a**) and PilA (**b**) homologues of *X. t.* pv. *graminis* Xtg29, *X. t.* pv. *arrhenatheri* LMG 727, *X. t.* pv. *poae* LMG 728, *X. t.* pv. *phlei* LMG 730, *X. t.* pv. *cerealis* CFBP 2541, *X. t.* pv. *undulosa* Xtu4699, *X. t.* pv. *translucens* DSM 18974, and *X. t.* DAR61454. Pathotype strains (^PT^) and the *X. translucens* type strain (^T^) are indicated. Colors of the alignments highlight conserved amino acids (*red*) and those with strongly similar properties (*green*). Within the conserved N-terminal region of PilA, a class III signal peptide was identified

Comparison of the non-canonical type III secretion system (T3SS) of the non-*graminis X. translucens* strains with the one identified in Xtg29, revealed significant differences in AA sequence identity (Fig. 6). High percentage sequence similarity was observed for 21 out of the 22 T3SS genes (AA identity >75%). In contrast, the HrpE pilin of the non-*graminis* strains revealed only 52.58 to 65.98% amino acid sequence identity when compared to Xtg29. A multiple sequence alignment unraveled further inter-pathovar differences of HrpE. We found less than 75% sequence identity of the corresponding homologues across the different *X. translucens* strains with the exceptions of LMG 727 and LMG 730 (96.91% AA identity), DSM 18974 and CFBP 2541 (97.94% AA identity), and DAR61454 and Xtu4699 (100% AA identity). The multiple sequence alignment further revealed the N-terminus as the most variable region, while the C-terminus was found to be largely conserved (Fig. 7a).

High sequence deviations were further found for pilins of the type IV pilus (T4P). Comparison of the different non-*graminis X. translucens* strains with the corresponding T4P genes in Xtg29, revealed two groups of genes, differing in conservation (Fig. 6). In total, 15 out of 22 genes were highly conserved with more than 94.49% sequence identity. In contrast, in the second group of T4P genes, significantly lower values were observed for the major pilin PilA (44.85–75.56%) and the minor pilins PilE, PilX, PilW, PilV, and FimT (35.98–52.41%). Additionally, we found the putative tip-associated adhesin PilY1 to share only 27.97 to 30.57% AA sequence identity with Xtg29. Similar to HrpE, also the major T4P pilin PilA revealed further distinct differences between the other *X. translucens* pathovars. Highest sequence similarity was observed for Xtu4699 and DAR61454 (99.34%) as well as LMG 728 and LMG 730 (97.3%), followed by 75.56% AA identity for the PilA homologues of LMG 727 and Xtg29. Otherwise, only low sequence similarity (40.85–64.58% AA identity) was found among the *X. translucens* pathovars. However, in contrast to HrpE, the PilA homologues were characterized by a highly conserved N-terminus of at least 28 AA, while the C-terminal region revealed extensive sequence deviations (Fig. 7b).

Analysis of the minor pilins (i.e. PilE, PilX, PilW, PilV, and FimT) and the putative tip-associated adhesin PilY1 revealed differing similarity patterns. Thus, *X. t.* pv. *graminis* Xtg29 and *X. t.* pv. *poae* LMG 728 revealed only 27.97 to 56.42 AA identity with any other *X. translucens* pathovar (Additional file 7: Figure S3), while those, in contrast, shared more than 74.55% AA sequence identity among each other. Among the seven *Xtg* strains, all pilins of the T4P and T3SS were highly conserved (AA identities >99%).

Comparison of the *graminis* group pathovars further revealed inter-pathovar differences of the *wxc* gene cluster of LPS O-antigen biosynthesis. These were manifested in a variable, central part of seven to nine genes, flanked by highly conserved regions of seven and six genes, respectively (Additional file 8, Figure S4). The variable gene cluster section was only found to differ between the pathovars *graminis*, *arrhenatheri*, *poae*, and *phlei,* but did not show intra-pathovar differences among the seven *Xtg* strains.

### A set of 74 CDS was specific to the *Xtg* core genome and revealed various pathogenicity-related functions

For comparison of the *Xtg* core genome with the non-*graminis X. translucens* strains, a set of 74 CDS was predicted as true singletons of the pv. *graminis*. Among those, 42 CDS encoded for hypothetical proteins including eight with signal peptide motifs (Additional file 9: Table S5). Additional two CDS showed homology to IS elements (i.e., one transposase and one integrase). The remaining 30 CDS were functionally annotated based on sequence similarity and conserved protein domains (Table 5). According to their predicted functions, 28 of those genes were assigned to four categories, i.e. nutrient acquisition, regulation & modification, virulence as well as adhesion & motility. Additionally, two genes encoding for an invertase and a putative ABC-type ATPase were grouped as genes of other functions. With ten representative genes, the group of nutrient acquisition formed one of the largest groups among the predicted singletons and was characterized by the prediction of seven secretion signals for SEC-dependent translocation across the cytoplasmic membrane. Members of the ‘nutrient acquisition group’ comprised two glycerophosphodiester phosphodiesterases as well as a TonB-dependent receptor and various transporters. Also the category ‘regulation & modification’ comprised ten CDS. Those largely encoded for restriction/modification systems as well as toxin-antitoxin components. Furthermore, we identified one transcriptional regulator of the XRE family as well as a peptidyl-prolyl cis-trans isomerase, further characterized by a predicted signal peptide. Four putative effector proteins were grouped into the category of virulence. Among those, only two (i.e. XTGART29_1903 and XTGART29_2268) revealed high sequence similarity (E-value < 10^−50^) to other *Xanthomonas* species. The remaining two stood out by their sequence identity to *Pseudomonas* spp., while also XTGART29_1903 possessed high sequence similarity to *Ralstonia solanacearum*. In total, three effector proteins were predicted to harbor an upstream plant-inducible-promoter (PIP) box. Another four CDS grouped in the category of adhesin and motility. Two neighboring CDS, which were separated by a sequence gap in the assembly data, exhibited sequence similarity to different parts of filamentous hemagglutinin. Specific gap closure of this sequence using Sanger sequencing revealed the presence of a transposase between the two CDS. Most striking was the prediction of the putative T4P adhesin PilY1 and the minor pilin PilX as singletons of *Xtg*, while corresponding homologues were also identified in the other pathovars. Closer manual inspection of these findings revealed that distinct PilY1 and PilX homologues with similarities of 30–40% exist for the corresponding CDS in the other pathovars. These, however, were discarded in the EDGAR-based similarity analysis due to the strict orthology cutoff as employed in the EDGAR framework.

**Table 5:** True singletons of the *X. t.* pv. *graminis* core genome identified in comparison with non-*graminis X. translucens* strains

Category^a^	Gene^b^	Description	Domain^c^	Feature^d^	Homology^e^
NA	0088	glycerophosphodiester phosphodiesterase	cd08566	S	X, O
NA	0362	putative phosphate ABC transporter	cl21456	S	X, O
NA	0369	biopolymer transporter ExbD	COG0848		
NA	0370	biopolymer transporter ExbB	COG0811	S	X, O
NA	0371	hemolysin activator protein	COG2831		X, O
NA	0436	TonB-dependent receptor	TIGR01782	S	X, O
NA	0438	putative MFS/sugar transport protein	pfam13347		X, O
NA	0441	putative glycerophosphodiester phosphodiesterase	cd08566	S	
NA	1321	MFS transporter	pfam07690		
NA	2335	alpha-galactosidase	cd14792	S	X, O
VI	0478	putative type III effector protein, XopE class	-	PIP	O
VI	0894	putative cysteine protease, YopT-like	cl04145		O
VI	1903	putative type III effector protein, XopJ class	cl07849	PIP	X, O
VI	2268	putative type III effector protein	-	PIP	X, O
RM	0087	peptidylprolyl isomerase	pfam00639	S	X
RM	0480	XRE family transcriptional regulator	COG1396		X, O
RM	1782	putative toxin-antitoxin system toxin component, VapC/FitB-like	cd09861		
RM	1783	putative toxin-antitoxin system antitoxin component, SpoVT/AbrB-like	smart00966		
RM	2588	type I restriction and modification protein subunit M	TIGR00497		X, O
RM	2592	putative type I restriction and modification protein subunit S	COG0732		O
RM	2593	type I restriction and modification protein subunit R	COG0610		X, O
RM	2620	toxin-antitoxin system toxin component, RelE/ParE family	COG3668		
RM	2990	site-specific DNA-methyltransferase	COG0863		O
RM	3368	C-5 cytosine-specific DNA methylase	COG0270		X, O
AM	0364	filamentous hemagglutinin, partial	-		X, O
AM	0365	filamentous hemagglutinin, partial	smart00912	S	X, O
AM	2129	type IV pilus assembly protein, tip-associated adhesin PilY1	COG3419		X, O
AM	2130	type IV pilus assembly protein PilX	COG4726		
OF	1784	invertase	cd03768		X, O
OF	2590	putative ABC-type ATPase	cl21455		

^a^ *Abbreviations*: *NA* nutrient acquisition, *VI* virulence, *RM* regulation and modification, *AM* adhesion and motility, *OF* other function

^b^ 4-digit number corresponding to the gene ID with the prefix XTGART29_. Listed genes were identified as true singletons of the *Xtg* core genome when compared against *X. t.* pv. *arrhenatheri* LMG 727, *X. t.* pv. *poae* LMG 728, *X. t.* pv. *phlei* LMG 730, *X. t.* pv. *cerealis* CFBP 2541, *X. t.* pv. *undulosa* Xtu4699, *X. t.* pv. *translucens* DSM 18974 and *X. t.* DAR61454

^c^ Conserved domains (E-value < 10^−06^)

^d^ Features describing the presence of an upstream PIP-box (PIP) or a signal peptide (S)

^e^ Homology to at least one other *Xanthomonas* species (X) or another bacterial genus (O; E-value < 10^−50^)

## Discussion

Comparative genome analysis was applied to unravel virulence-contributing traits of *Xanthomonas translucens* pv. *graminis* (*Xtg*) with a link to its pathogenicity on Italian ryegrass. Our results clearly demonstrate that the *Xtg* strains form a genetically distinct group characterized by a large conserved core genome. Comparison to other *X. translucens* pathovars allowed the identification of pathovar *graminis*-specific genomic traits involved in regulatory mechanisms and nutrient acquisition. Furthermore, distinct amino acid sequences of putative pathogen-associated molecular patterns (PAMPs), such as pilins of the non-canonical type III secretion system and the type IV pilus were found to distinguish *Xtg* strains from other *X. translucens* pathovars and might influence host adaptation of the pv. *graminis*.

### *Xtg* strains represent a genetically distinct *X. translucens* pathovar

The close genetic relation of *Xtg* strains is reflected by a large core genome and a phylogenetic clustering distinct from the other *X. translucens* pathovars. Interestingly, we also found that all seven *Xtg* strains possessed only small numbers of strain-specific singletons, although the pathotype strain CFBP 2053 genome revealed roughly 300 additional CDS in comparison to the genome data of *Xtg* strains sequenced in this study. However, our data showed that only the singletons of CFBP 2053 comprised transposases, for which several identical paralogues were predicted across the genome. Genomic regions with transposase genes are generally difficult to assemble, because they often occur in identical copies across the genome [45]. In contrast to the *Xtg* strains sequenced in this study, the SOAP GapCloser [46] has been used to close gaps in the genome assembly of CFBP 2053 [47]. This may explain at a technical level the observations of transposase genes in CFBP 2053, where they represent the main cause for the higher number of predicted CDS.

### Swimming-deficiency is a common characteristic of *Xtg* strains

The absence of a flagellar gene cluster as found for the majority of *Xtg* strains analyzed in this study as well as the observed swimming-deficiency on soft agar confirmed the formerly reported non-motility of the pathovar *graminis* [48] (Fig. 4). Yet, the presence of a nearly complete flagellar gene cluster in the swimming-deficient *Xtg* strain NCPPB 3709 suggests that also motile pv. *graminis* strains may exist. Although, to the best of our knowledge, none has been described so far. The flagellum has been reported to primarily function in epiphytic biofilm formation and to enable the bacteria to move towards suitable entry points [49]. As *Xtg* is primarily transmitted by contaminated mowing tools [50], flagellar motility seems not essential for the pathogen’s lifestyle. The same transmission strategies are likely to be valid among all forage grass affecting *X. translucens* pathovars, such as pv. *arrhenatheri*, pv. *poae*, and pv. *phlei*, of which all pathotype strains, however, were found to be capable of swimming. Aside from mediating bacterial motility, the flagellum features one of the most prevalent and best studied pathogen-associated molecular patterns (PAMP), i.e. the flagellin peptide flg22, which is an important elicitor of plant defense response [8]. Therefore, absence of the flagellar locomotion machinery might favor *Xtg*’s ability to cause bacterial wilt on a wide host range by avoiding the elicitation of plant defense by flagellin.

### The T6SS is not a prevalent virulence factor of the pv. *graminis*

In contrast to the other *Xtg* strains, Xtg2 was found to harbor all 13 homologues of a type VI secretion system (T6SS), recently described to represent the T6SS core genes [51]. When compared to T6SS gene clusters identified in other *Xanthomonas* spp., the one identified in Xtg2 revealed highest sequence similarity to a cluster recently described to be specific to cereal affecting xanthomonads such as *X. translucens* DAR61454 [18]. Due to the secretion of effector proteins and toxins, the T6SS was found to contribute to virulence and bacterial competition of bacterial pathogens [52]. However, high virulence on Italian ryegrass was also observed for *Xtg* strains lacking the T6SS, indicating that it may be neither an essential nor a prevalent virulence factor of the pv. *graminis*.

### Intra-pathovar differences in virulence on Italian ryegrass are likely to come from strain-specific characteristics

Testing of the different *Xtg* strains for their pathogenicity on Italian ryegrass (*Lolium multiflorum*) revealed significant differences between the highly virulent Swiss strains and those isolated in Norway (NCPPB 3709) and New Zealand (ICMP 6431; Fig. 3). Both, NCPPB 3709 and ICMP 6431 were isolated from *L. perenne*, while the Swiss strains originated from *L. multiflorum* (Table 1). Thus, it is tempting to speculate that differences in virulence might be dependent on different host adaptation. Previous studies on *Xtg* strains, however, revealed no differences in virulence due to varying host plants or different geographical origin [4]. Furthermore, we did not find any CDS to be shared within, but not among the two groups and suppose differences in strain-specific traits to be causative for the observed variations. These may be related to certain genes and/or single nucleotide polymorphisms (SNPs) in coding sequences or regulatory elements. For the Norwegian *Xtg* strain NCPPB 3709 for instance, we observed a nonsense mutation in the xanthan biosynthesis gene cluster, which resulted in the fusion of *gumK* and *gumL*. It has been found for *X. oryzae* that mutation of *gumK* has resulted in a reduction of both, xanthan production and lesion length in a susceptible rice cultivar [53]. Thus, the gene fusion in the gene cluster of NCPPB 3709 may affect production of the extracellular polysaccharide xanthan and its pathogenicity on Italian ryegrass. However, in accordance with a recent finding for *X. t.* pv. *arrhenatheri* LMG 727, where a fusion of *gumK* and *gumL* was also observed [16], the mucoid appearance of NCPPB 3709 showed no differences in comparison to the other *X. translucens* strains.

### Differences in the LPS O-antigen encoding *wxc* gene cluster differentiate *Xtg* from other *graminis*-group pathovars

Lipopolysaccharides (LPS) are the main component of the bacterial cell envelope of Gram-negative bacteria and are well-known elicitors of plant defense reactions such as the release of reactive oxygen species and accumulation of pathogenesis related proteins [9]. Analysis of the seven *Xtg* strains revealed slight intra-pathovar differences within the *wxc* gene cluster, which is involved in the LPS O-antigen biosynthesis [54]. These may only cause minor differences on the LPS structure, as previous profiling revealed a homogeneous LPS pattern within the pv. *graminis* [55]. The study further reported the pv. *graminis* LPS profile to differ from the other *X. translucens* pathovars. Recent comparative genome analyses revealed inter-pathovar differences of the *wxc* gene cluster for *X. t.* pv. *arrhenatheri*, *X. t.* pv. *poae*, and *X. t.* pv. *phlei* [16]. These differences derive from a region within the *wxc* gene cluster consisting of seven to eight genes, which also revealed distinct differences to *X. t.* pv. *graminis*. Among the investigated *Xtg* strains, however, the corresponding region was well conserved (Additional file 8: Figure S4). Mutational analysis of the *wxc* gene cluster in *X. campestris* pv. *campestris* led to the differentiation of three regions, involved in the LPS O-antigen biosynthesis, the formation of the LPS core as well as the translocation and modification of sugar composition [54]. Thus, differences in the *wxc* gene cluster observed for the *Xtg* strains and among the *X. translucens* pathovars may affect different structural components. Hypervariability of the LPS gene cluster is common among *Xanthomonas* spp., but revealed no apparent correlation with host specificity [56]. Nevertheless, intra-species differences as observed for *X. translucens* might result in pathovar-specific LPS structures, which so far have not been experimentally resolved.

### *Xtg* strains possess distinct primary structures for pilins of the T4P and T3SS

As described for other plant pathogenic bacteria, *in planta* colonization by *Xtg* is likely to be dependent on the type IV pilus (T4P), which mediates adherence and twitching motility [57]. A corresponding gene cluster was also identified in the other *X. translucens* pathovars. While most of the homologues were well conserved, we observed highly divergent primary structures for the T4P pilin subunits PilA, PilE, PilX, PilV, PilW and FimT of *X. t.* pv. *graminis* (Fig. 6). With respect to the direct contact with the host organism, it seems likely that the T4P pilus subunits may be elicitors of plant defense. Sequence variations as observed for the *X. translucens* pathovars may therefore facilitate their ability to cause bacterial wilt in certain host plants by evading recognition through the plant defense machinery [58]. As recently reviewed by Dunger et al. [59], the T4P plays an important role in pathogenesis of various *Xanthomonas* species. The contribution of the type IV pilus for bacterial wilt in forage grasses has not been addressed so far, but the largely conserved gene cluster indicates that it may assist plant colonization. If that is the case, especially the high sequence variability of the pilin subunits and its putative role in host adaptation are worth deeper analysis.

Also, the type III secretion system represents an extracellular structure, which mediates direct contact to the host organism [60]. Comparison of different *X. translucens* pathovars revealed a largely conserved gene cluster of the non-canonical type III secretion system as previously described for Xtg29 [12]. The pilus subunit HrpE, in contrast, displayed strong sequence heterogeneity. The sequence deviations mainly occurred in the N-terminal sequence, while the C-terminus was largely conserved among the *X. translucens* pathovars (Fig. 7). Similar findings were previously reported for comparative analysis of HrpE homologues in other *Xanthomonas* species [60, 61]. The authors proposed the highly variable N-terminus as the surface exposed region, while the C-terminus most likely encodes for the polymerization domain of HrpE. The C-terminal region of *X. translucens* HrpE homologues revealed only low amino acid identity to the investigated xanthomonads (Additional file 10: Figure S5). However, hydrophobicity plot analysis by using the Kyte and Doolittle hydrophobicity scale [62] revealed a similar pattern as observed for the other Hrp pilins and therefore suggests a similar folding [63] (data not shown). Moreover, it has been reported, that the knock-out of *hrpE* in Xtg29 resulted in a drastically reduced virulence on Italian ryegrass as also observed for mutants of the regulator HrpG and the structural component HrcR [12]. With respect to these findings, we assume HrpE as a functional subunit for type III secretion and the observed differences in percentage sequence identity as pathovar-specific traits.

### *Xtg*-specific CDS are characterized by effector proteins, transporters, regulatory systems and a large number of hypothetical proteins

More than half of the 74 CDS predicted to be specific to *X. translucens* pv. *graminis*, were predicted as hypothetical proteins. Among those, 18 were found to be conserved in other bacteria and therefore suggest that they may be essential for bacterial fitness [64]. Moreover, eight were predicted to possess a signal peptide, which indicates that they are involved in the interaction with the environment [65]. Several studies revealed a reduced or even complete loss of virulence for hypothetical proteins affected by transposon-insertion mutagenesis in *Xanthomonas* spp. [66, 67]. Thus, along with the functionally annotated CDS, the identified hypothetical proteins need to be considered as crucial pathogenicity-associated traits of *X. t.* pv. *graminis*.

Among the functional annotated CDS, four type III effector proteins were predicted to be specific to the pv. *graminis* core genome. Three of those revealed a plant-inducible promoter (PIP) sequence, which indicates that they are regulated in HrpX-dependent manner as part of the HrpX regulon [68]. Moreover, the effector proteins were largely found to reveal high sequence similarity to other bacterial genera such as *Pseudomonas* and *Ralstonia*, which might indicate their acquisition by horizontal gene transfer [69]. This assumption is further supported by the low GC values of 53.24 to 60.22%, which deviate considerably from the *Xtg* average of 69% (Additional file 1: Table S1). Moreover, we found both, an integrase and a transposase downstream of XTGART29_1903. These genes were also predicted as true singletons of the *Xtg* core genome. With view on the host range determining function of effector proteins [70], it is likely that the four effector proteins contribute to the virulence of *Xtg*.

Among the other *Xtg*-specific CDS, nine signal peptides were predicted. These are likely to be targeted to the Sec system, which mediates the translocation over the inner membrane into the periplasm and is in some cases linked to a second translocation step into the extracellular milieu via the type II secretion system (T2SS) [71]. Effectively, we identified various transporters along with a TonB-dependent receptor, which are embedded in the bacterial cell wall and mediate the uptake of phosphate, iron-siderophore complexes, vitamin B12 and carbohydrates [72]. The importance of such transporter systems for successful host colonization has been demonstrated by a transposon insertion mutagenesis study, which revealed a reduction of both, the *in planta* multiplication and the virulence of *X. albilineans* [67].

With view on the pathogen’s lifestyle beyond plant invasion, i.e. epiphytic and/or saprophytic growth, the transcriptional regulation of virulence factors is crucial for energy conservation, evasion of host defense and disease development [73]. Recent transcriptome analysis revealed differential gene expression of T2SS substrates not only in dependence of the cultivation media but also between the two *X. citri* pv. *citri* strains XccA306 and Xcaw12879, which are characterized by broad host range and high host specificity, respectively [74]. Thus, it is possible that regulatory mechanisms play a central role for host adaptation processes of *X. t.* pv. *graminis*. Genes involved in ‘regulation and modification’ formed the second largest group of functional annotated singletons of the pv. *graminis* and largely comprised components of restriction-modification and toxin-antitoxin complexes. Restriction-modification systems are mainly known for their protective function against foreign DNA from phages [75]. Both, the DNA methyltransferase (modification domain) and the restriction enzyme (restriction domain) recognize the same target sequence and solely DNA lacking the modification imprint are degraded by the endonuclease. The type I restriction system identified within the *Xtg* singletons is a multifunctional enzyme consisting of three different domains, which are important for the methylation and restriction function [75]. The two orphan methyltransferases (MTases), in contrast, did not reveal a neighboring restriction enzyme and are most likely involved in epigenetic regulation [76]. Among others, DNA MTases were shown to affect motility, adherence, and virulence [77] and thus represent promising regulators of virulence mechanisms in *X. t.* pv. *graminis*.

Toxin-antitoxin systems are also part of the bacterial epigenetic regulation [78]. Co-transcription and co-translation of the stable toxin and degradation-prone antitoxin secures ordered segregation of replicons prior to cell division and plasmid maintenance due to post-segregationally killing [79]. The toxin-antitoxin (TA) system identified within the *Xtg* singletons revealed similarity to TA system recently described to be a common characteristic of various xanthomonads [80]. Additionally, a ParE-like toxin was found to be specific to the *Xtg* core genome. The corresponding antitoxin component, which was also conserved among the *Xtg* strains, revealed a homologue in the genome of *X. t.* pv. *cerealis* CFBP 2541 and was therefore not predicted as singleton. With view on the recently demonstrated virulence and avirulence function of the type III secreted effector and TA toxin AvrRxoI in *Xanthomonas* spp. [81], it would be interesting to elucidate the functional role of the identified TA system components in the *Xtg* – *L. multiflorum* interaction.

## Conclusion

Our study substantially increased the available *Xtg* genome data by making results from additional strains available. This data was utilized for comparative genomics that also considered previously supplied genomes of the species *X. translucens*. In combination with a phenotypic characterization of key features, we identified genomic loci related to effector proteins, regulatory mechanisms, and nutrient acquisition as putative virulence contributing characteristics that distinguish *Xtg* from non-*graminis X. translucens* strains. Host adaptation of *Xtg* might be facilitated by the identified, unique primary structures conceivably involved in evasion of the plant perception machinery. These genomic traits represent promising candidates for new functional research with the ultimate aim to better understand fundamental principles of bacterial host adaptation and facilitate targeted breeding of new forage grass cultivars for bacterial wilt resistance.

## Additional files

Additional file 1: Table S1. Sequencing data of *Xtg* strains. The results were obtained by sequencing of single paired-end libraries only (i.e. Xtg2, Xtg9, Xtg10, NCPPB 3709, and ICMP 6431) or in combination with a mate-pair library (i.e. Xtg29) by the Illumina MiSeq System. (DOCX 16 kb)
Additional file 2: Table S2. Xtg29 genome statistics based on data of the 454 Titanium FLX System and the Illumina MiSeq System sequencing strategies. (DOCX 15 kb)
Additional file 3: Figure S1. Number of CDS shared by and unique to *X. t.* pv. *graminis* of different geographic origin. The two Venn diagrams represent the comparison of either the core genome of the Swiss strains, i.e. Xtg29, Xtg2, Xtg9, and Xtg10 (A) or the Norwegian (NCPPB 3709) and New Zealand (ICMP 6431) strain (B) against the other *Xtg* strains. (PDF 299 kb)
Additional file 4: Figure S2. Number of strain-specific CDS predicted for seven *Xtg* strains. The data reflect the amount of predicted hypothetical proteins (blue), CDS with annotated functions (green) as well as detected transposases (grey). (PDF 171 kb)
Additional file 5: Table S3. Type VI secretion system homologues identified in Xtg2 and their corresponding COG numbers (E-value < 10^−20^). Except of two CDS (i.e. XTGART2_1599 and XTGART2_1602), homologues were also identified in the genomes of Xtg9, Xtg10, and NCPPB 3709. (DOCX 15 kb)
Additional file 6: Table S4. Type IV pilus homologues identified for Xtg29 and their corresponding COG numbers (E-value < 10^−12^). (DOCX 17 kb)
Additional file 7: Figure S3. Percentage identity matrix of the minor type IV pilus pilins PilE, PilX, PilW, PilV, and FimT as well as the T4P adhesin PilY1 across *X. translucens* strains (i.e. *X. t.* pv. *graminis* Xtg29, *X. t.* pv. *arrhenatheri* LMG 727, *X. t.* pv. *poae* LMG 728, *X. t.* pv. *phlei* LMG 730, *X. t.* pv. *cerealis* CFBP 2541, *X. t.* pv *undulosa* Xtu4699, *X. t.* pv. *translucens* DSM 18974, and *X. t.* DAR61454) including pathotype strains (^PT^) and the *X. translucens* type strain (^T^). Class III signal peptides identified for the minor pilins are indicated. For PilW homologues in *X. t.* pv. *translucens* DSM 18974 and *X. t.* DAR61454 no corresponding class III signal peptide was found. (PDF 386 kb)
Additional file 8: Figure S4.
*wxc* gene cluster comparison of four *X. translucens* pathovars represented by *X. t.* pv. *graminis* Xtg2 and the pathotype strains *X. t.* pv. *arrhenatheri* LMG 727^PT^, *X. t.* pv. *poae* LMG 728^PT^, and *X. t.* pv. *phlei* LMG 730^PT^. The strain Xtg2 was chosen as a representative of the pv. *graminis*. Intra-pathovar differences of *Xtg* strains, i.e. CDS with gene separation events due to non-sense mutations as observed for Xtg29, ICMP 6431, and NCPPB 3709, are indicated by red borders. Inter-pathovar gene cluster comparison revealed a highly variable region highlighted by a grey background, while the flanking regions were largely conserved. (PDF 202 kb)
Additional file 9: Table S5.
*Xtg* core singleton CDS which encode hypothetical proteins or IS elements. (DOCX 17 kb)
Additional file 10: Figure S5. Multiple sequence alignment of HrpE homologues of *Xanthomonas* spp. including seven *Xanthomonas translucens* pathovars. (PDF 51 kb)
Additional file 11: Figure S6. Topology of the phylogenetic tree of *X. translucens* pathovars including percent conservation values in 500 bootstrapping iterations. (PDF 343 kb)

## Abbreviations

COG: Clusters of orthologous groups
CTAB: Cetyltrimethylammonium bromide
IS: Insertion
*Rpf*: Regulation of pathogenicity factors
*Xps*: *Xanthomonas* protein secretion
