# Supplementary material for: Comparative genomics of host adaptive traits in Xanthomonas translucens pv. graminis
Source: BMC Genomics. 2017 Jan 5;18:35. doi: 10.1186/s12864-016-3422-7 (PMC5217246; doi:10.1186/s12864-016-3422-7)
Supplement: Additional file 1: Table S1. — Sequencing data of Xtg strains. The results were obtained by sequencing of single paired-end libraries only (i.e. Xtg2, Xtg9, Xtg10, NCPPB 3709, and ICMP 6431) or in combination with a mate-pair library (i.e. Xtg29) by the Illumina MiSeq System. (DOCX 16 kb) [file 12864_2016_3422_MOESM1_ESM.docx]

**Additional file 1: Table S1. Sequencing data of *Xtg* strain.** The results were obtained by sequencing of single paired-end libraries only (i.e. Xtg2, Xtg9, Xtg10, NCPPB 3709, and ICMP 6431) or in combination with a mate-pair library (i.e. Xtg29) by the Illumina MiSeq System.

| **Attribute** | **Xtg29^1^** | **Xtg2** | **Xtg9** | **Xtg10** | **NCPPB 3709** | **ICMP 6431** |
| --- | --- | --- | --- | --- | --- | --- |
| **Library** | Mate pair + paired end | Paired end | Paired end | Paired end | Paired end | Paried end |
| **Aligned reads (All/PE)** | 3885865/1509084 | 2050174/962649 | 3839824/1801806 | 2057089/960847 | 2463810/1176088 | 2060064/962860 |
| **Assembled bases** | 837318256 | 479884384 | 979194020 | 521799192 | 554729581 | 524564778 |
| **PE-size(s)** | 588 +/- 195; 5701 +/- 1458 | 518 +/- 187 | 546 +/- 194 | 536 +/- 199 | 481 +/- 182 | 574 +/- 213 |
| **Coverage** | 185 | 115 | 235 | 125 | 131 | 127 |
| **GC content (%)** | 69 | 69 | 69 | 69 | 69 | 69 |
| **Scaffolds** | 3 | 296 | 288 | 290 | 288 | 284 |
| **Contigs (> 500 bp)** | 369 | 365 | 367 | 364 | 363 | 349 |
| **N50 scaffold length** | 1476948 | 18819 | 19988 | 19914 | 20011 | 20035 |
| **N50 contig length** | 18109 | 18385 | 18554 | 18404 | 18840 | 18459 |

^1^ The assembly data for Xtg29 are derived from the combination of sequencing data of a paired-end and a mate-pair library
