# Supplementary material for: Comparative genomics of host adaptive traits in Xanthomonas translucens pv. graminis
Source: BMC Genomics. 2017 Jan 5;18:35. doi: 10.1186/s12864-016-3422-7 (PMC5217246; doi:10.1186/s12864-016-3422-7)
Supplement: Additional file 2: Table S2. — Xtg29 genome statistics based on data of the 454 Titanium FLX System and the Illumina MiSeq System sequencing strategies. (DOCX 15 kb) [file 12864_2016_3422_MOESM2_ESM.docx]

**Additional file 2: Table S2. Xtg29 genome statistics based on data of the 454 Titanium FLX System and the Illumina MiSeq System sequencing strategies.**

| **Attribute** | **Xtg29**  **(Illumina MiSeq)** | **Xtg29**  **(454 FLX System)** |
| --- | --- | --- |
| **Genome size (bp)** | 4150581 | 4100864 |
| **Contigs (> 500 bp)** | 369 | 788 |
| **DNA scaffolds** | 3 | 12 |
| **Total genes** | 3543 | 3619 |
| **Genes in internal clusters** | 825 | 801 |
| **Genes with function prediction** | 2573 | 2501 |
