# Supplementary material for: Comparative genomics of host adaptive traits in Xanthomonas translucens pv. graminis
Source: BMC Genomics. 2017 Jan 5;18:35. doi: 10.1186/s12864-016-3422-7 (PMC5217246; doi:10.1186/s12864-016-3422-7)
Supplement: Additional file 3: Figure S1. — Number of CDS shared by and unique to X. t. pv. graminis of different geographic origin. The two Venn diagrams represent the comparison of either the core genome of the Swiss strains, i.e. Xtg29, Xtg2, Xtg9, and Xtg10 (A) or the Norwegian (NCPPB 3709) and New Zealand (ICMP 6431) strain (B) against the other Xtg strains. (PDF 299 kb) [file 12864_2016_3422_MOESM3_ESM.pdf]

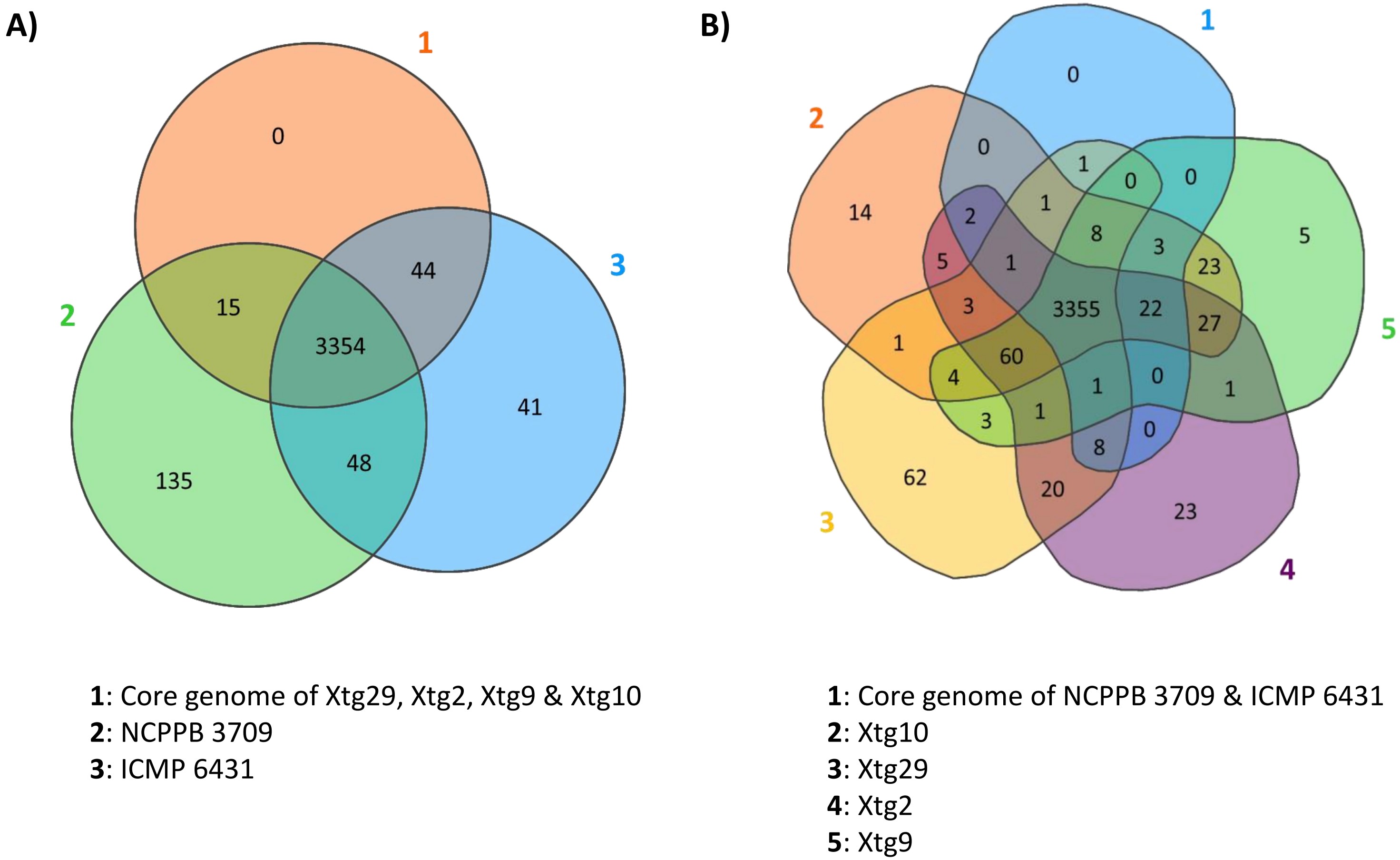

Additional file 3: Figure S1. Number of CDS shared by and unique to *X. t.* pv. *graminis* of different geographic origin. The two Venn diagrams represent the comparison of either the core genome of the Swiss strains, i.e. Xtg29, Xtg2, Xtg9, and Xtg10 (A) or the Norwegian (NCPPB 3709) and New Zealand (ICMP 6431) strain (B) against the other *Xtg* strains.
