# Supplementary material for: Comparative genomics of host adaptive traits in Xanthomonas translucens pv. graminis
Source: BMC Genomics. 2017 Jan 5;18:35. doi: 10.1186/s12864-016-3422-7 (PMC5217246; doi:10.1186/s12864-016-3422-7)
Supplement: Additional file 4: Figure S2. — Number of strain-specific CDS predicted for seven Xtg strains. The data reflect the amount of predicted hypothetical proteins (blue), CDS with annotated functions (green) as well as detected transposases (grey). (PDF 171 kb) [file 12864_2016_3422_MOESM4_ESM.pdf]

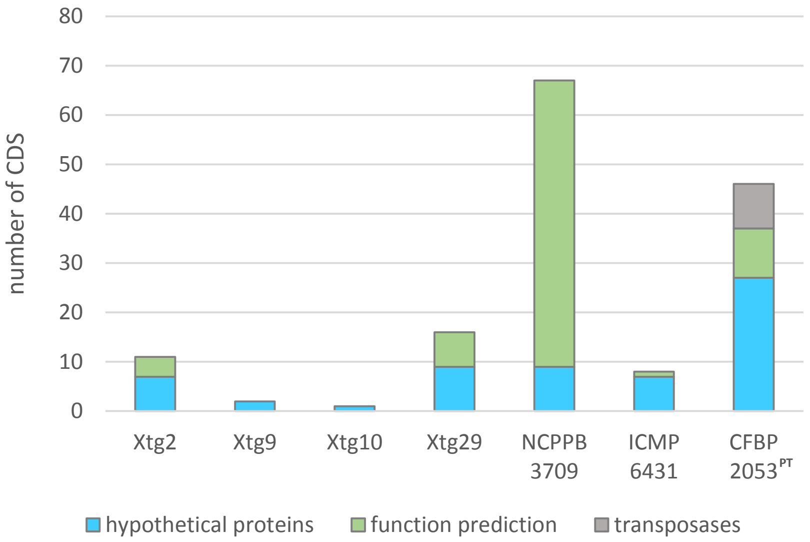

Additional file 4: Figure S2. Number of strain-specific CDS predicted for seven *Xtg* strains. The data reflect the amount of predicted hypothetical proteins (blue), CDS with annotated functions (green) as well as detected transposases (grey).
