# Supplementary material for: Comparative genomics of host adaptive traits in Xanthomonas translucens pv. graminis
Source: BMC Genomics. 2017 Jan 5;18:35. doi: 10.1186/s12864-016-3422-7 (PMC5217246; doi:10.1186/s12864-016-3422-7)
Supplement: Additional file 5: Table S3. — Type VI secretion system homologues identified in Xtg2 and their corresponding COG numbers (E-value < 10−20). Except of two CDS (i.e. XTGART2_1599 and XTGART2_1602), homologues were also identified in the genomes of Xtg9, Xtg10, and NCPPB 3709. (DOCX 15 kb) [file 12864_2016_3422_MOESM5_ESM.docx]

| **Gene ID** | **Gene description** | **Domain** |
| --- | --- | --- |
| XTGART2_1599 | type VI secretion system protein | COG3515 |
| XTGART2_1602 | type VI secretion system protein | COG3523 |
| XTGART2_2320 | type VI secretion system protein | COG3455 |
| XTGART2_2321 | type VI secretion system protein | COG3522 |
| XTGART2_2322 | type VI secretion system lipoprotein | COG3521 |
| XTGART2_2324 | type VI secretion system protein | COG3516 |
| XTGART2_2325 | type VI secretion system protein | COG3517 |
| XTGART2_2326 | type VI secretion system protein | COG3157 |
| XTGART2_2327 | type VI secretion system protein | COG3518 |
| XTGART2_2328 | type VI secretion system protein | COG3519 |
| XTGART2_2329 | type VI secretion system protein | COG3520 |
| XTGART2_2330 | type VI secretion system ATPase | COG0542 |
| XTGART2_2332 | type VI secretion system protein | COG3501 |

**Additional file 5: Table S3. Type VI secretion system homologues identified in Xtg2 and their corresponding COG numbers (E-value < 10^-20^).** Except of two CDS (i.e. XTGART2_1599 and XTGART2_1602), homologues were also identified in the genomes of Xtg9, Xtg10 and NCPPB 3709.
