# Supplementary material for: Comparative genomics of host adaptive traits in Xanthomonas translucens pv. graminis
Source: BMC Genomics. 2017 Jan 5;18:35. doi: 10.1186/s12864-016-3422-7 (PMC5217246; doi:10.1186/s12864-016-3422-7)
Supplement: Additional file 6: Table S4. — Type IV pilus homologues identified for Xtg29 and their corresponding COG numbers (E-value < 10−12). (DOCX 17 kb) [file 12864_2016_3422_MOESM6_ESM.docx]

**Additional file 6: Table S4. Type IV pilus homologues identified for Xtg29 and their corresponding COG numbers (E-value < 10^-12^)**

| **Name** | **Gene ID** | **Gene description** | **Domain** |
| --- | --- | --- | --- |
| PilM | XTGART29_0848 | type IV pilus assembly protein PilM | COG4972 |
| PilN | XTGART29_0849 | type IV pilus assembly protein PilN | COG3166 |
| PilO | XTGART29_0850 | type IV pilus assembly protein PilO | COG3167 |
| PilP | XTGART29_0851 | type IV pilus assembly protein PilP | COG3168 |
| PilQ | XTGART29_0852 | type IV pilus secretin PilQ | COG4796 |
| PilD | XTGART29_0961 | prepilin signal peptidase PilD | COG1989 |
| PilC | XTGART29_0962 | putative type IV pilus assembly protein PilC | COG1459 |
| PilA | XTGART29_0963 | type IV pilus assembly protein, major pilin PilA | COG4969 |
| PilB | XTGART29_0968 | type IV pilus assembly ATPase PilB | COG2804 |
| PilR | XTGART29_0971 | putative two-component system response regulator PilR | COG2204 |
| PilS | XTGART29_0972 | putative two-component system sensor protein PilS | COG3852 |
| FimX | XTGART29_2080 | GGDEF and EAL domain containing protein | COG2200 / COG2199 |
| PilE | XTGART29_2128 | type IV pilus assembly protein PilE | COG4968 |
| PilY1 | XTGART29_2129 | type IV pilus assembly protein, tip-associated adhesin PilY1 | COG3419 |
| PilX | XTGART29_2130 | type IV pilus assembly protein PilX | COG4726 |
| PilW | XTGART29_2131 | type IV pilus assembly protein PilW | COG4966 |
| PilV | XTGART29_2132 | type IV pilus assembly protein PilV | COG4967 |
| FimT | XTGART29_2133 | type IV pilus assembly protein FimT | COG4970 |
| PilZ | XTGART29_2634 | type IV pilus assembly protein PilZ | COG3215 |
| PilU | XTGART29_2361 | twitching motility protein PilU | COG5008 |
| PilT | XTGART29_2362 | twitching motility protein PilT | COG2805 |
| PilF | XTGART29_1848 | type IV pilus assembly protein PilF | COG3063 |
