# Supplementary material for: Comparative genomics of host adaptive traits in Xanthomonas translucens pv. graminis
Source: BMC Genomics. 2017 Jan 5;18:35. doi: 10.1186/s12864-016-3422-7 (PMC5217246; doi:10.1186/s12864-016-3422-7)
Supplement: Additional file 7: Figure S3. — Percentage identity matrix of the minor type IV pilus pilins PilE, PilX, PilW, PilV, and FimT as well as the T4P adhesin PilY1 across X. translucens strains (i.e. X. t. pv. graminis Xtg29, X. t. pv. arrhenatheri LMG 727, X. t. pv. poae LMG 728, X. t. pv. phlei LMG 730, X. t. pv. cerealis CFBP 2541, X. t. pv undulosa Xtu4699, X. t. pv. translucens DSM 18974, and X. t. DAR61454) including pathotype strains (PT) and the X. translucens type strain (T). Class III signal peptides identified for the minor pilins are indicated. For PilW homologues in X. t. pv. translucens DSM 18974 and X. t. DAR61454 no corresponding class III signal peptide was found. (PDF 386 kb) [file 12864_2016_3422_MOESM7_ESM.pdf]

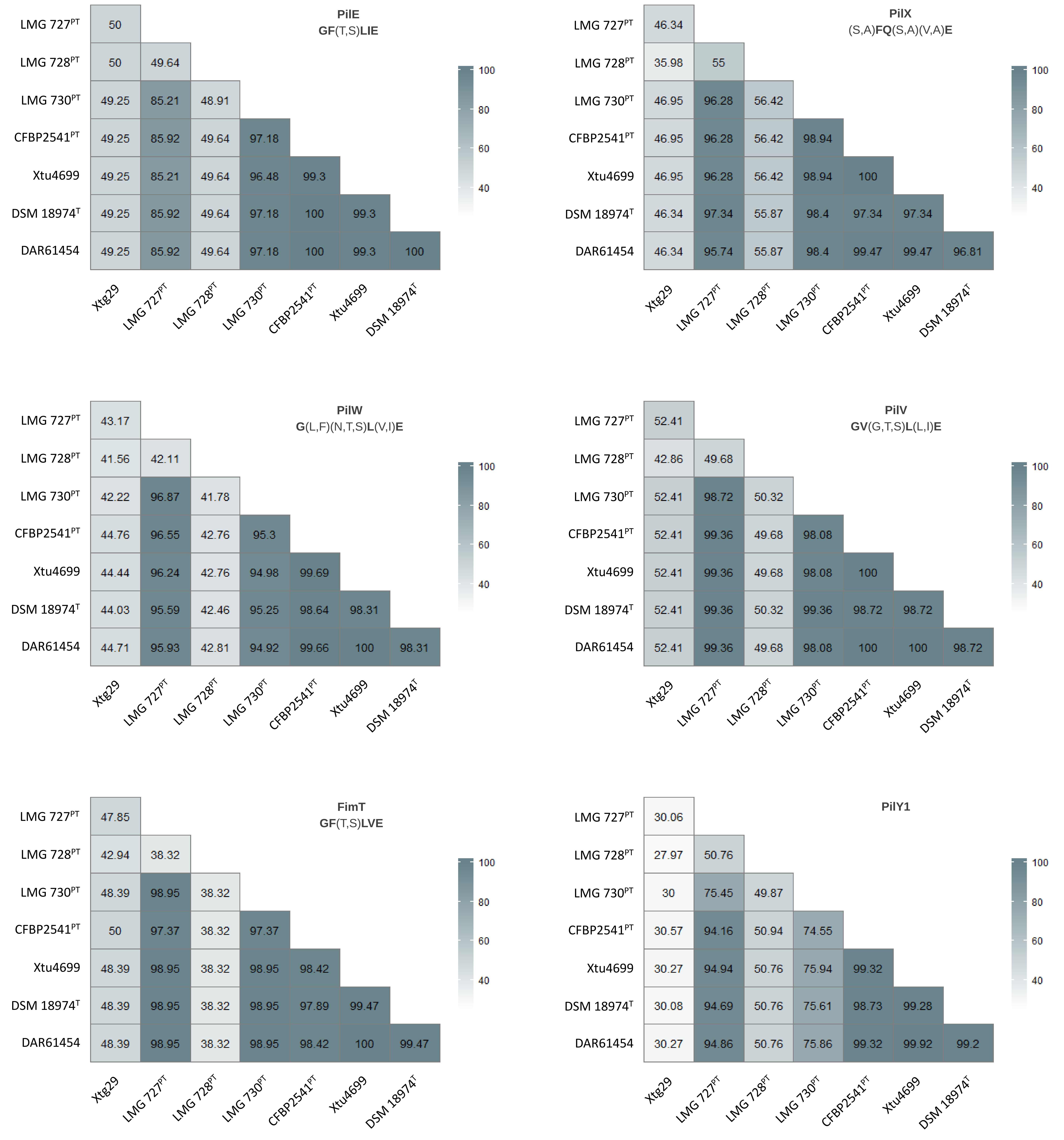

Additional file 7: Figure S3. Percentage identity matrix of the minor type IV pilus pilins PilE, PilX, PilW, PilV, and FimT as well as the T4P adhesin PilY1 across eight *X. translucens* strains (i.e. *X. t.* pv. *graminis* Xtg29, *X. t.* pv. *arrhenatheri* LMG 727, *X. t.* pv. *poae* LMG 728, *X. t.* pv. *phlei* LMG 730, *X. t.* pv. *cerealis* CFBP 2541, *X. t.* pv *undulosa* Xtu4699, *X. t.* pv. *translucens* DSM 18974, and *X. t.* DAR61454) including pathotype strains (^{PT}) and the *X. translucens* type strain (^{T}). Class III signal peptides identified for the minor pilins are indicated. For PilW homologues in *X. t.* pv. *translucens* DSM 18974 and *X. t.* DAR61454 no corresponding class III signal peptide was found.
