# Supplementary material for: Comparative genomics of host adaptive traits in Xanthomonas translucens pv. graminis
Source: BMC Genomics. 2017 Jan 5;18:35. doi: 10.1186/s12864-016-3422-7 (PMC5217246; doi:10.1186/s12864-016-3422-7)
Supplement: Additional file 8: Figure S4. — wxc gene cluster comparison of four X. translucens pathovars represented by X. t. pv. graminis Xtg2 and the pathotype strains X. t. pv. arrhenatheri LMG 727PT, X. t. pv. poae LMG 728PT, and X. t. pv. phlei LMG 730PT. The strain Xtg2 was chosen as a representative of the pv. graminis. Intra-pathovar differences of Xtg strains, i.e. CDS with gene separation events due to non-sense mutations as observed for Xtg29, ICMP 6431, and NCPPB 3709, are indicated by red borders. Inter-pathovar gene cluster comparison revealed a highly variable region highlighted by a grey background, while the flanking regions were largely conserved. (PDF 202 kb) [file 12864_2016_3422_MOESM8_ESM.pdf]

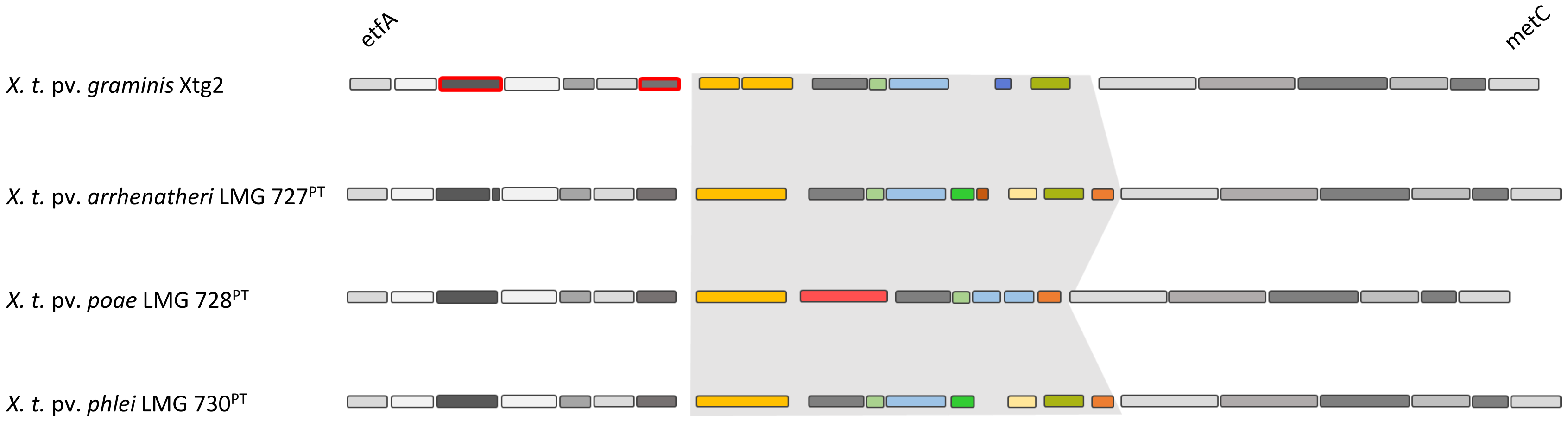

Additional file 8: Figure S4. *wxc* gene cluster comparison of four *X. translucens* pathovars represented by *X. t.* pv. *graminis* Xtg2 and the pathotype strains *X. t.* pv. *arrhenatheri* LMG 727^{PT}, *X. t.* pv. *poae* LMG 728^{PT}, and *X. t.* pv. *phlei* LMG 730^{PT}. The strain Xtg2 was chosen as a representative of the pv. *graminis*. Intra-pathovar differences of *Xtg* strains, i.e. CDS with gene separation events due to non-sense mutations as observed for Xtg29, ICMP 6431, and NCPPB 3709, are indicated by red borders. Inter-pathovar gene cluster comparison revealed a highly variable region highlighted by a grey background, while the flanking regions were largely conserved.
