# Supplementary material for: Comparative genomics of host adaptive traits in Xanthomonas translucens pv. graminis
Source: BMC Genomics. 2017 Jan 5;18:35. doi: 10.1186/s12864-016-3422-7 (PMC5217246; doi:10.1186/s12864-016-3422-7)
Supplement: Additional file 9: Table S5. — Xtg core singleton CDS which encode hypothetical proteins or IS elements. (DOCX 17 kb) [file 12864_2016_3422_MOESM9_ESM.docx]

**Additional file 9: Table S5. *Xtg* core singleton CDS which encode hypothetical proteins or IS elements.**

| **Gene ID** | **Description** | **Feature^1^** | **Homology^2^** |
| --- | --- | --- | --- |
| XTGART29_0086 | hypothetical protein |  | X |
| XTGART29_0111 | hypothetical secreted protein |  |  |
| XTGART29_0151 | hypothetical protein |  |  |
| XTGART29_0171 | hypothetical protein | S | X, O |
| XTGART29_0361 | hypothetical protein |  |  |
| XTGART29_0366 | hypothetical protein | S |  |
| XTGART29_0367 | conserved hypothetical protein | S | X, O |
| XTGART29_0368 | hypothetical protein |  |  |
| XTGART29_0435 | hypothetical protein | S | O |
| XTGART29_0439 | hypothetical protein |  |  |
| XTGART29_0440 | hypothetical protein |  |  |
| XTGART29_0442 | hypothetical protein |  |  |
| XTGART29_0481 | hypothetical protein |  | X, O |
| XTGART29_0951 | hypothetical protein | S |  |
| XTGART29_1034 | hypothetical protein | S | X, O |
| XTGART29_1069 | hypothetical protein |  |  |
| XTGART29_1449 | hypothetical protein |  |  |
| XTGART29_1533 | hypothetical protein |  |  |
| XTGART29_1644 | conserved hypothetical protein |  | X, O |
| XTGART29_1645 | conserved hypothetical protein |  | X |
| XTGART29_1646 | conserved hypothetical protein |  | X |
| XTGART29_1647 | conserved hypothetical protein |  | X |
| XTGART29_1885 | hypothetical protein |  |  |
| XTGART29_1894 | hypothetical protein |  | O |
| XTGART29_1895 | hypothetical protein |  | O |
| XTGART29_1902 | hypothetical protein |  | X |
| XTGART29_1973 | hypothetical protein |  |  |
| XTGART29_1979 | hypothetical protein |  |  |
| XTGART29_2046 | hypothetical protein |  |  |
| XTGART29_2127 | hypothetical protein |  |  |
| XTGART29_2346 | hypothetical protein |  |  |
| XTGART29_2347 | hypothetical protein |  | X |
| XTGART29_2348 | hypothetical protein |  | X, O |
| XTGART29_2589 | hypothetical protein |  |  |
| XTGART29_2591 | hypothetical protein |  | X, O |
| XTGART29_2733 | hypothetical protein | S | X |
| XTGART29_2803 | hypothetical protein |  |  |
| XTGART29_2912 | hypothetical protein |  |  |
| XTGART29_3229 | hypothetical protein | S |  |
| XTGART29_3261 | hypothetical protein |  |  |
| XTGART29_3280 | hypothetical protein |  | X |
| XTGART29_3529 | hypothetical protein |  | X |
| XTGART29_1904 | integrase |  | X, O |
| XTGART29_1905 | transposase |  | X, O |

^1^ Features describing the presence of a signal peptide (S).

^2^ Homology to at least one other *Xanthomonas* species (X) or another bacterial genus (O; E-value < 10^-50^).
