# Supplementary material for: Comparative genomics of host adaptive traits in Xanthomonas translucens pv. graminis
Source: BMC Genomics. 2017 Jan 5;18:35. doi: 10.1186/s12864-016-3422-7 (PMC5217246; doi:10.1186/s12864-016-3422-7)
Supplement: Additional file 10: Figure S5. — Multiple sequence alignment of HrpE homologues of Xanthomonas spp. including seven Xanthomonas translucens pathovars. (PDF 51 kb) [file 12864_2016_3422_MOESM10_ESM.pdf]

```
X. translucens pv. graminis Xtg29              MPIPFGGLANMGSAATAISSLTNGLAGVGISTASGMANNAIGAGQMGGTLATMQGDVANQ
X. translucens pv. undulosa Xtu4699            MSFPFSALGHGSSTGIAAGSFGMGLKDLGISGLSGMGNNLIAGGQMGGMIGNMRTNVSEQ
X. translucens DAR61454                        MSFPFSALGHGSSTGIAAGSFGMGLKDLGISGLSGMGNNLIAGGQMGGMIGNMRTNVSEQ
X. translucens pv. arrhenatheri LMG727PT       MALPFSSLSNAYGASTIGSSFGQGLSGLGLSAASGMGNNAIAAGQMGGMVATMGANVKDQ
X. translucens pv. phlei LMG730PT              MALPFSSLSNAYGASTIGSSFGSGLSGLGLSAASGMSNNAISAGQMGGMVATMGANVKDQ
X. translucens pv. poae LMG728PT               MPFPSGLIGSGYSAYTAAGYFGQGLMNLGISTAAGMGNNAIGAGQMAGMVGNMRADVSNQ
X. translucens pv. cerealis CFBP2541PT         MALPFSALANAGSASTAFGSFRNGLAGLGINGASGMANNAIGAGQMGCMVGNMRTDVSNQ
X. translucens pv. translucens DSM18974T       MALPFSALANAGSASTAFGSFRNGLAGLSINGASGMANNAIGAGQMGSMVGNMRTDVSNQ
X. campestris pv. campestris ATCC 33913T       ---MLNLQSIVPRLGQARDLLGSDLSS-RFDNHTATQT---SDNQMDSLMGGIGKSAAAQ
X. euvesicatoria AAD21326                      MQIFPEVSSWRSRVGQGMDCFTGGLSN-GISGAAALSG---ANGQMDSLLGDMSASDEAQ
X. oryzae pv. oryzae MAFF311018                MEILPQISSLNSRFQQGMDGYTGGVAN-GISGASALSG---SNGQMGSLLGDMSASDEAQ
X. axonopodis pv. citri 306                    MELLPQISSIKSRFDQGTDAYTGGVSG-GISGEAALTG---ANGQMSSLISDMNASDEAQ
X. axonopodis pv. glycines 8ra                 MELFPQISSLNSRFEQGMDGYTGGVSG-GISGADALSG---ANGQMSSLISDMTASDEAQ
                                                       .         .    .: .  :.   .      . .**   :. :  .   *

X. translucens pv. graminis Xtg29
X. translucens pv. undulosa Xtu4699            EAMMEAVTRLQNELNFKAAECNLAKQAGQNVKSLTQG-
X. translucens DAR61454                        EAMMDQVTALQNELNFHMAMDSLAKQAGSNAKQLTSGS
X. translucens pv. arrhenatheri LMG727PT       EAMMDQVTALQNELNFHMAMDSLAKQAGSNAKQLTSGS
X. translucens pv. phlei LMG730PT              EAMMDQVTQMQNELNMHMAMDQLAKQAGANAKSLTQG-
X. translucens pv. poae LMG728PT               EAMMDQVTQMQNELNMHMAMDQLAKQAGANAKSLTQG-
X. translucens pv. cerealis CFBP2541PT         EAMMDQVTQMQNELNMHMAMDQLSKQAGANAKSLTQG-
X. translucens pv. translucens DSM18974T       EAMMDQVTQMQNELNMHMAMCQLSKQAGANAKSLTQG-
X. campestris pv. campestris ATCC 33913T       EAMMDQVTQMQNELNMHMAMCQLSKQAGANAKSLTQG-
X. euvesicatoria AAD21326                      ERMNNYLTAKKNELDFNVALNKFIGKAGDNAKQLVGQ-
X. oryzae pv. oryzae MAFF311018                KSMNNKITMLKNDLDFNVALNKFIGKAGDNAKQLVGQ-
X. axonopodis pv. citri 306                    KSMNNKITQLKNDLDFNVALNKFIGKAGDNAKQLVGQ-
X. axonopodis pv. glycines 8ra                 KSMNNKITQLKNDLDFNVALNKFIGKAGDNAKQLVGQ-
                                               KSMNNKITMLKNDLDFNVALNKFIGKAGDNAKQLVGQ-
                                               : * : :*  :*:*::: *  .:  :** *.*.*.
```

Additional file 10: Figure S5. Multiple sequence alignment of HrpE homologues of *Xanthomonas* spp. including seven *Xanthomonas translucens* pathovars.
