# Supplementary material for: Comparative genomics of host adaptive traits in Xanthomonas translucens pv. graminis
Source: BMC Genomics. 2017 Jan 5;18:35. doi: 10.1186/s12864-016-3422-7 (PMC5217246; doi:10.1186/s12864-016-3422-7)
Supplement: Additional file 11: Figure S6. — Topology of the phylogenetic tree of X. translucens pathovars including percent conservation values in 500 bootstrapping iterations. (PDF 343 kb) [file 12864_2016_3422_MOESM11_ESM.pdf]

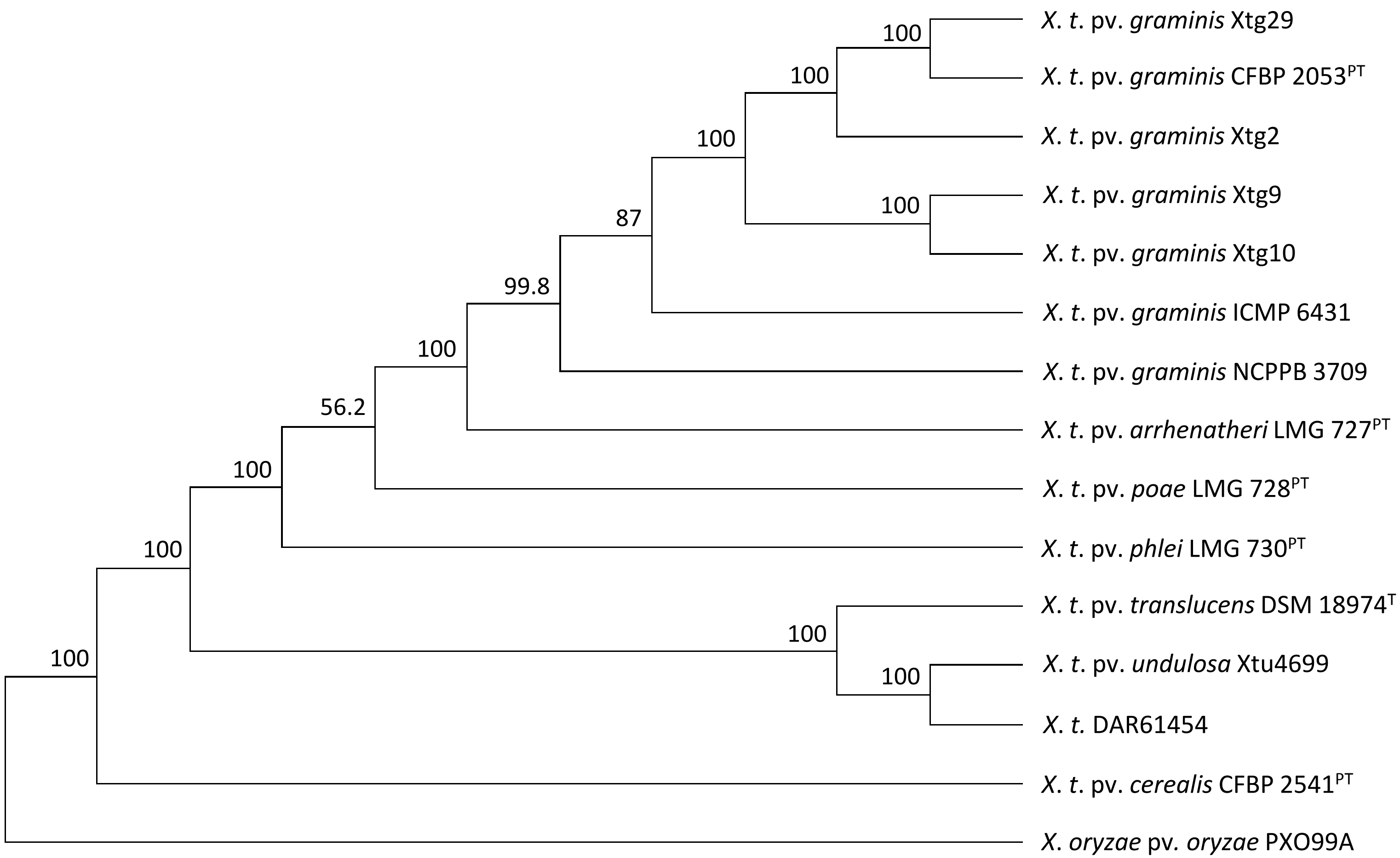

Additional file 11: Figure S6. Topology of the phylogenetic tree of *X. translucens* pathovars including percent conservation values in 500 boostrapping iterations.
